# Intrinsic Properties of tRNA Molecules as Deciphered via Bayesian Network and Distribution Divergence Analysis

**DOI:** 10.3390/life8010005

**Published:** 2018-02-08

**Authors:** Sergio Branciamore, Grigoriy Gogoshin, Massimo Di Giulio, Andrei S. Rodin

**Affiliations:** 1 Department of Diabetes Complications and Metabolism, Diabetes and Metabolism Research Institute, City of Hope, Duarte, 91010 CA, USA; ggogoshin@coh.org; 2 Early Evolution of Life Laboratory, Institute of Biosciences and Bioresources, CNR, 80131 Naples, Italy; massimo.digiulio@ibbr.cnr.it

**Keywords:** tRNA identity, tRNA recognition, operational code, bayesian networks, information theory, distribution divergence

## Abstract

The identity/recognition of tRNAs, in the context of aminoacyl tRNA synthetases (and other molecules), is a complex phenomenon that has major implications ranging from the origins and evolution of translation machinery and genetic code to the evolution and speciation of tRNAs themselves to human mitochondrial diseases to artificial genetic code engineering. Deciphering it via laboratory experiments, however, is difficult and necessarily time- and resource-consuming. In this study, we propose a mathematically rigorous two-pronged in silico approach to identifying and classifying tRNA positions important for tRNA identity/recognition, rooted in machine learning and information-theoretic methodology. We apply Bayesian Network modeling to elucidate the structure of intra-tRNA-molecule relationships, and distribution divergence analysis to identify meaningful inter-molecule differences between various tRNA subclasses. We illustrate the complementary application of these two approaches using tRNA examples across the three domains of life, and identify and discuss important (informative) positions therein. In summary, we deliver to the tRNA research community a novel, comprehensive methodology for identifying the specific elements of interest in various tRNA molecules, which can be followed up by the corresponding experimental work and/or high-resolution position-specific statistical analyses.

## 1. Introduction

A prototypical tRNA is a compact and ubiquitous molecule. Its primary function is that of an adapter mediating mRNA and growing protein sequence; as such, together with aminoacyl tRNA synthetases (aaRSes), tRNAs form the backbone of translation apparatus and embody the genetic code. In order to be effective, translation machinery requires unambiguous charging of tRNA molecules by the appropriate amino acids (“aa”); this, somewhat whimsically, is known as “second genetic code” (see [1] and references therein). It is the aaRSes that are physically responsible for charging tRNAs with amino acids.

What are the factors that determine the identity of tRNAs with respect to these pairings? How, exactly, is the “correct” tRNA recognized and aminoacetylated by cognate aaRS? It is reasonable to assume that this specificity is encoded in the tRNA molecule proper (perhaps localized around certain tRNA regions) and is then reflected in its 3D structure and chemical properties. Identifying such determinants and antideterminants (that, conversely, prevent incorrect recognition) within tRNA molecules is far from trivial, yet is of significant theoretical and practical importance (see [2] for an overview).

The straightforward approach to elucidating tRNA determinants and antideterminants would be via experimental work. This constitutes primarily combinatorial screening, where many tRNA mutants are checked for interaction with aaRSes. Specific combinations of nucleotide polymorphisms that correlate with changes in interaction status of tRNAs with cognate aaRSes are then noted. Intense experimental effort along the above lines led to identifying some such tRNA sequence features that are therefore assumed to be key elements of the “second genetic code” ([2]). These experimental efforts are ongoing ([3,4] and references therein). It should also be noted that, gratifyingly, our understanding of the underlying chemistry of amino acid activation is improving ([5,6]); recent progress in molecular dynamic simulations had also added to our insight into the tRNA – aaRS fundamental biology (see [7] for an overview). On a side note, much research had been done on the variability in the aaRSes themselves ([8,9]). This has substantial practical implications (see Datt and Sharma [10] for an overview of disease-associated aaRS mutation spectra).

However, our general grasp of the tRNA “determinant array” is very fragmented, at best. We can only speculate as to the completeness, universality and robustness of discovered determinants (single and multiple tRNA nucleotide polymorphisms) within the “second genetic code” context. To put it bluntly, we lack systemic approach to the problem.

Experimental approaches are very time- and resource-consuming. While “targeted evolution” of both tRNAs and aaRSes is technologically feasible ([11,12]) and happens to be of much practical importance (see [13] for a mt-tRNA mitochondrial multi-system disorder example), the direction in which such experimental work is pursued is often arbitrarily, if not anecdotally, mapped out.

Here, we propose a novel in silico approach for inferring important tRNA positions and features. Our methodology of choice is Bayesian (or Belief) Network (BN) modeling, which has been used by us ([14,15]) and others before in the domains with similar data structure. This methodology is augmented by estimation of the distribution divergence metrics for the tRNA positions, across different tRNA subclasses (subgroups), an information-theoretic approach. The primary data is a compendium of tRNA nucleotide sequences (of which there are many thousands presently available), coupled with both biological/chemical information (crystallographic data, biochemical behavior, etc.) and ontological data (existing large body of work reflected in recent literature on various aspects of tRNA chemistry, biology, evolution, and significance in general).

Our two main goals throughout this study were to (i) further improve the BN methodology (including both algorithms and software), predominantly in testing its generalization to novel data sets and types; and to (ii) gain better tools for identifying tRNA determinants (and studying tRNA evolution in general). The former is largely outside of the scope of this communication; we will concentrate on the latter. As our approach was purely data-driven, the rationale was to infer “interesting” (the most structurally important) positions/features of tRNAs regardless of the biological context. However, we have also followed up with the discussion of the discovered positions of interest using ontological (literature) knowledge. It should be noted that, as always in biology (and especially in the case of tRNAs), any “feature of interest” exists largely in the evolutionary sense, which was a constant consideration throughout the analyses and discussion of the results presented here.

In this study, we used a systems biology/probabilistic inference methodology (BNs) that in our opinion is well suited for the task; it is detailed elsewhere ([14,15]), but the rationale is briefly covered below in Materials and Methods. By applying BN analysis to the tRNA sequence data, we were able to recognize the interplay of structurally and biologically important features of the tRNA molecule. Furthermore, by extending the analysis to distribution divergence estimates across all tRNA positions and different tRNA subclasses, we were able to single out the important positions specific to each type of tRNA, which led us to identifying tRNA determinants and antideterminants.

## 2. Materials and Methods

Building tRNA probabilistic networks, as well as evolutionary studies of tRNAs in general, require assembling a reliable tRNA sequence alignment as the initial step. Resulting BNs are dependent on both the alignment and the number of sequences involved; more accurate alignment and higher number of sequences being of course desirable. In this study, we went to the tRNAdb database as a primary source ([16]); 9758 unambiguously aligned tRNA sequences were imported.

**Figure 1:**
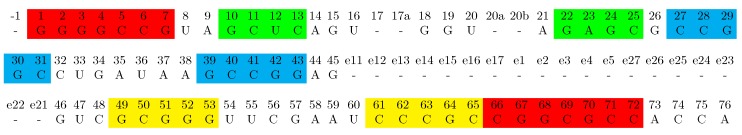
tRNA sequence alignment. The first row corresponds to the “standard” numbering scheme. The second row is the consensus sequence. Structural parts of tRNA molecules are highlighted in color (see text for details).

Each vertical position in the alignment is interpreted as a random variable with values ranging over all possible nucleotide states at this position in the tRNAs in the alignment. In other words, each vertical position is a discrete random variable with a number (label) assigned. These labels, or variable names, are thus invariant for all tRNA molecules. To avoid confusion with position numbering, Figure 1 depicts the layout of consensus tRNA sequence. The first row corresponds to the numbering scheme adopted throughout most of this study; position numbers in the first row are also the variable (node) names in the BNs. This is a universally accepted tRNA numbering standard ([17]). The master, or majority consensus, tRNA sequence (second row in Figure 1) contains notable fragments of tRNA molecule colored in red (acceptor stem), green (D-arm), blue (anticodon 131 arm) and yellow (T-arm). The same color scheme is used in the resulting BNs. Therefore, by consulting Figure 1 when evaluating these study results, it is easy to visually link to the specific positions and sections of the tRNA molecule.

Given the tRNA alignment, a BN, also formally known as PDAG (probabilistic directed acyclic graph), can be learned from it. Dependencies (intra-tRNA position interactions represented by the BN edges; they can also be colloquially thought of as “correlations” or “linkage” or “associations” between positions) suggested by the BN can subsequently be evaluated against a set of well-known structural features of tRNA. The purpose of doing so (building the BN and observing significant dependencies, as reflected by the network edges) is two-fold: to identify yet unknown but potentially interesting interactions, and to observe deviations from the established models. In a tRNA BN, network nodes correspond to the variables representing vertical positions in tRNA alignment, edges—to the dependencies between these variables. For a fuller understanding of BN representation, notions of conditional independence, edge directionality and strength, BN equivalence classes, and Markov neighborhood (or “blanket”) of a node are useful; however, detailed theoretical treatment is outside the scope of this communication (see [15,18,19]). Briefly, BN is a graphical model of a joint multivariate probability distribution of random (both continuous and discrete) variables that reflects relationships of dependence and conditional independence among them. Absence of an edge between two nodes (variables) indicates conditional independence. Directionality of the edge (arrow) is, in this (tRNA) case, for mathematical convenience only and does not imply causation flow. Dependency (edge) strength can also be estimated; it should be interpreted as a relative support for the strength of a relationships between the variables. No absolute interpretation, such as *p*-value, is possible before the follow-up parametric statistical analysis; however, edge strengths can be directly compared to one another within a BN. Edge strengths are shown as numbers (next to the corresponding edges in the BN figures) that can be thought of as the likelihood ratios (marginal likelihood given the data, or model fit, of the BN with the edge present divided by the marginal likelihood of the BN with the edge absent). Local probability tables (conditional probabilities) can be estimated for pairwise frequency calculations between the two nodes; by observing these frequencies, we can, for example, speculate on the nature of different nucleotide pairings at different tRNA positions (e.g., whether they are canonical Watson–Crick pairings or not).

Parsing local probability tables can become cumbersome for the nodes with more than one or two edges; the problem is exacerbated when conditioning by the amino acid (or other tRNA subgroup stratification) is introduced. This is why we have also decided to complement the BN modeling with the simpler information theoretic-based pairwise analyses of the tRNA sequence data conditioned on the tRNA subclass variables, notably the amino acid variable. BN modeling remains the method of choice for higher-order abstraction; for scrutinizing tRNA position relationships at a finer level (and in smaller, for example stratified by amino acid, tRNA datasets), such pairwise comparisons (utilizing distribution divergence) might be more practical and statistically robust.

It remains to note that the actual BN search algorithm used by us was an iterative gradient method with restarts, and MDL (Minimum Description Length) was chosen as a model scoring criterion. Multinomial local probability model with four nucleotide states was used. Further technical details of our current BN modeling implementation can be found in [15,20]. Various BN reconstruction algorithm parameter adjustments were tried based on our previous experience with BNs (results not shown); the resulting BNs proved to be sufficiently robust. Our BN modeling software is directly available from the authors.

## 3. Results

### 3.1. Structural Information

**Figure 2:**
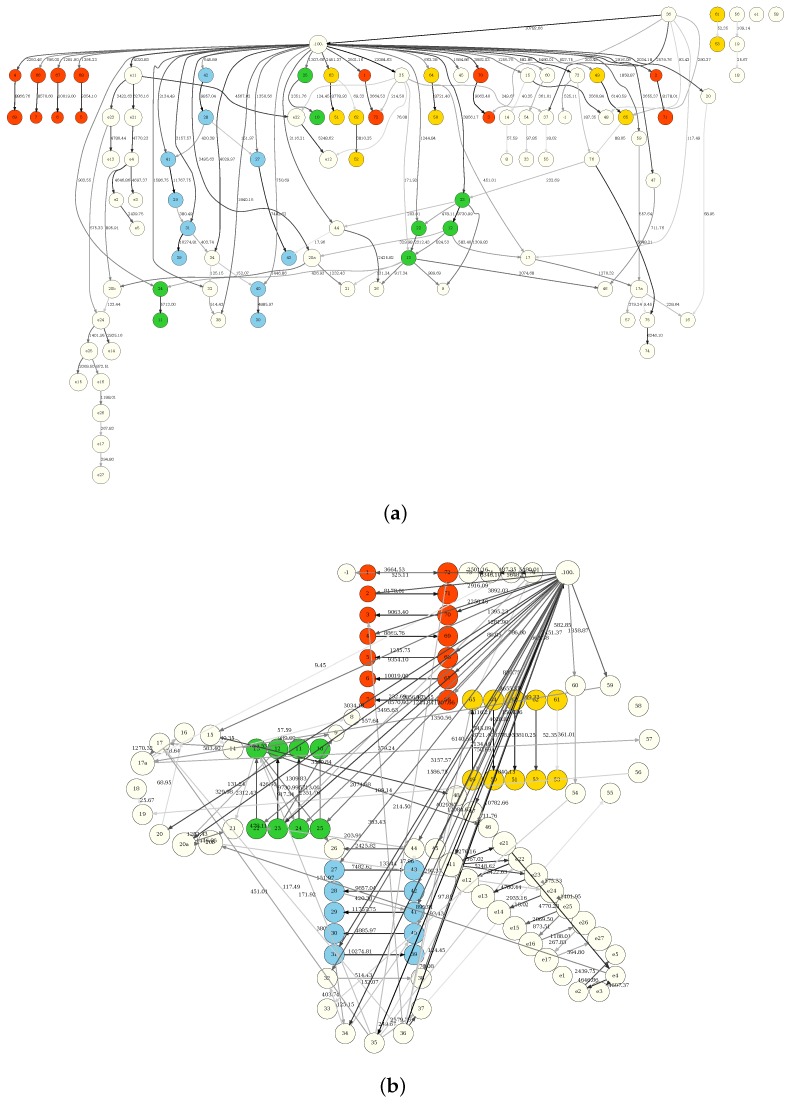
Bayesian network built from the full set of tRNA sequences. (**a**) direct visualization of the PDAG (Probabilistic Directed Acyclic Graph); (**b**) same, superimposed on the secondary tRNA structure. Nodes in the network correspond to the variables (specifically, tRNA positions, as enumerated in Figure 1, first row), and edges to the dependencies between the variables. “Boldness” of the edge is proportional to the dependency strength, also indicated by the number shown next to the edge. See text for BN construction details. *(Label “100” does not refer to a tRNA position but rather is a placeholder for the cognate aa variable, appearing here for the technical convenience reasons only; directionality of the edge (arrow) is for mathematical convenience reasons only as well, and does not imply causation.)*

The complete tRNA BN (using all tRNAdb sequences pooled together) is shown in Figure 2. Figure 2a is the “flattened” visualization that makes it easier to trace specific position dependencies. Figure 2b shows the same graph superimposed on the cloverleaf tRNA structure, for easier “biological” visualization. The interpretation of the dependencies (links, edges) between the tRNA positions in the network is necessarily equivocal—a dependency might have different (one or more) biological interpretations. Some of these dependencies, such as the ones directly explained by chemical bonds and physical interactions, are more straightforward and obvious. However, even these can be partially obscured by the probabilistic nature of BN reconstruction.

We will first consider the dependencies related to the secondary structure of tRNA. For example, in the acceptor stem, positions 1, 7 are linked with positions 72, 66, respectively; positions 10, 13 are linked with 25, 22 in D-arm; positions 27, 31 are linked with 43, 39 in anticodon arm; positions 49, 53 are linked with positions 66, 61 in the T-arm. These connections are expected and provide a solid point of reference (and a positive control, especially if one takes into account relative connection strengths) for further BN interpretation. Clearly, the BN modeling is able to identify structural relationships inherent to the secondary cloverleaf structure. At this point, we were curious if the procedure was also capable of suggesting principal tertiary structure interactions (known from the literature). The following relationships proved to be of particular interest:

First, note the interaction between position 26 and 44. These are often associated with a non-Watson–Crick pairing (usually GA, with the last base change from 33 degree to 45 degree angle).

Then, a parallel base pair interaction is indicated between position 15 in the d-loop and position 48 in the variable loop (bringing the D-loop and the variable loop together in the 3D configuration). A reverse Hoogsteen pairing is indicated with position 8 and position 14; it is known that this interaction, together with positions 15 and 48, is responsible for the linkage of the two main domains of tRNA molecule in the l-shaped configuration via precise docking of the D- and T psi C-arms). It should be noted that, although all four of these positions are largely invariant (or semi-invariant), the BN methodology is able to elucidate their relationships and suggest how all of them are linked together in maintaining the l-shaped structures of tRNA molecules. This is not surprising, considering that BN analysis is very sensitive to rare events ([15,20]. The latter are often ignored altogether in traditional statistical analyses (as well as in common classification algorithms, such as single decision trees in machine learning). Indeed, once a *p*-value (or, correspondingly, a decision tree classification decision) is generated, the rare event is simply no longer visible in the analysis schema; however, BN reconstruction process often creates an additional (comparatively weak) edge instead of just disregarding the “minority report” of a rare event. In such cases, low confidence in the edge (reflected as relatively low edge strength, as measured by the model marginal likelihood ratio) points to a rarity of the event (a particular tRNA mutation, in this context) rather than the statistical insignificance of the relationship.

Additionally, a triple base interaction is suggested between positions 9, 23 and 13. Another triple base interaction between 13, 22 and 46 is related to docking the variable loop onto the D-arm. However, another important interaction occurs between the D- and C-loops (link between G18G19 of D-loop with position 55c56). The BN shows the interactions between positions 18, 19, and 56, but not position 55. All of these relationships, highlighted by the BN, are essential to the determination of the core tertiary structure of the tRNA molecule. This is gratifying in itself; however, even a superficial glance at the Figure 2 reveals many more potential relationships in addition to what has been so far reported in the literature (which largely revolves around the intricacies of the tertiary structure determination and interactions).

Before following up on these potential relationships, however, one question that must be asked is whether the BN “overfits” the data (i.e., suggests spurious relationships reflecting random noise and data analysis artifacts). We have studied the question extensively in the broader BN modeling context ([14,15]). By using entropy-based model selection (and BN scoring) criteria, we can explicitly adjust the overfitting/underfitting balance (i.e., penalize less/more for the network complexity). The BN reconstruction engine used throughout this study was tuned more towards “underfitting”, meaning that if the edge appears in the BN visualization, our confidence in it is sufficiently high (in other words, “if the edge is present, there is definitely something”). What the BN reconstruction doe, is simply underscores, agnostically, all information patterns contained in the data. Therefore, in our analyses of the results shown in Figure 2, it would behoove us to go beyond secondary and tertiary structure interpretations.

For example, consider the interplay between the positions 9, 12, 13, 22, 23 and 46. The “triple” interaction between 9, 27, 23 and 13, 22, 46 has been reported before. However, in addition to that, we observe a more complex pattern of interactions that hinges on position 9. We can say that position 9 is dependent on the state of position 13 (or 22, which is tightly linked to 13) and position 23 (or 12, which is tightly linked to 23). Which of these alternatives reflects the actual biological (mechanistic) relationship? We cannot tell from the BN alone; all we know is that the likelihood of 9-13 is slightly higher than that of 9-22 (and the likelihood of 9-23 is slightly higher than 9-12). However, this might be a sampling artifact. However, in any case, the BN suggests that there is a “global” pattern of interaction between all five nucleotides. Further untangling of the underlying biology would be an example of promising direction of laboratory research. One possible interpretation is that the crystal structures, in general, point to one specific configuration (the crystallized form) whereas in solution more than one tRNA structure can dynamically exist. tRNAs are flexible polymers, and what we see depicted in the BN are the multiple interactions that occur during small structural rearrangements of the molecule in vivo (and recall that BN modeling is particularly revealing of rare events/variants). These can reflect the fluctuations around the minimum free energy structure (often but not always the crystallized form)—or the phenomenon could be due to the alternative structures related to specific biological functions (e.g., conformational changes due to interaction with other molecules, such as ribosomes or aaRSes). Either way, more laboratory research is definitely indicated for this particular position cluster.

In general, it is our opinion that one could look at the tRNA molecule as a system (of interactions)—if so, the BN (such as in Figure 2) is a synthetic representation of this system, where a change in one base pair would affect numerous other positions either due to direct chemical interactions, or because it will alter the global stability of the molecule.

To illustrate this outlook, let us revisit the interplay of the positions 12, 13, 22 and 23 at the far end of the d-arm close to the d-loop. The links between positions 12, 23 and 13, 22 are what we expect to see with the d-arm formation in the canonical cloverleaf tRNA structure. Interpretation of the connections 12, 13 and 22, 23, however, is not immediately obvious. A closer inspection of the BN, local probability tables, and tRNA alignments reveals that positions 13, 22 do not always form a canonical Watson–Crick (GU) pairing; sometimes, it is instead the two adenine residues that cannot pair. This means that the d-domain, in a subset of the tRNA molecules, presents a shorter arm and a larger loop. Analysis of the BN local probability tables reveals that in order to accommodate this structural variation (with a shorter and therefore less stable arm) positions 12, 23 have to simultaneously change from AT to the higher energy GC, to maintain the sufficient structure stability.

In other words, what the BN analysis is suggesting here (with the connections 12, 13 and 22, 23) are the constraints in the evolutionary dynamics of the d-domain of tRNA molecule. A local change in the sequence (and therefore in the structural configuration) must be physically and thermodynamically compatible with the biological structure and cannot take place unless the molecular “neighbors” are ready to accommodate the change event. Thus, the BN points us in the direction of these “neighbors”, revealing the pattern of nucleotides changes in the linked positions. The non-canonical structure (with a larger loop and a shorter arm in the D-domain) is the results of one of the possible evolutionary paths of the tRNA molecule: these paths are reflected in the concerted changes in positions 12, 13, 22 and 23. One such evolutionary scenario, compatible with the observed BN, is as follows: initial mutations occur at positions 13, 22 (AU to GC). This event does not alter the secondary structure (it could be considered a neutral event), but represents a “pre-adaptation” to the next events that lead to the breakage of the stem at positions 12, 23 bringing about the altered secondary structure with a larger loop and a shorter stem.

While Figure 2 shows the BN built from all the available tRNA sequence data combined, obviously similar BNs can be constructed from the different subgroups (or (sub)classes; we will use these terms interchangeably throughout the manuscript) of tRNAs. Such subclasses can be stratified by the species groups, domains of life, amino acids, etc. *(Series of aa-stratified BNs are available directly from the authors, in pdf or dot formats.)* Then, one can compare resulting BNs by observing topological differences (presence or absence of particular edges between the nodes). Qualitative and quantitative analysis of these topological differences can prove very useful in elucidating various tRNA determinants. Such detailed analysis is outside of the scope of the present study (however, see the following two sections for the baseline analyses of amino acid- and domain of life-based stratifications), but it is our intention to carry out comprehensive tRNA subgroup analyses in the future, predominantly to clarify certain aspects of tRNA gene histories. One caveat here, though, is the necessarily smaller samples, leading to the less robust BNs. For such smaller samples, "downgrading" from high-level-abstraction of graphical multivariate analysis (BNs, in their essence) to series of statistically rigorous pairwise tests might be advantageous. In the following section, we describe precisely such an approach.

### 3.2. Amino Acids Specificity

As depicted in Figure 2, the full-set tRNA BN is reflective of the overall organization of tRNA molecules, comprising all position associations that can be extracted from the data (inclusive of all types of tRNAs). Therefore, some of the observed relationships are common to all (or most of) the tRNAs present in the database, whereas others apply only to selected subsets. For example, in a group of tRNAs specific for a given amino acid, a singular base (or a specific combination of bases) could be primarily responsible for amino acid determination by aaRSes.

Here, we set out to investigate the positional relationships specific to particular classes of amino acids. Simply put, this analysis utilizes the notion that if the position *i* is, in some sense, “important” for a specific class of tRNAs, the conditional distribution of nucleotides at *i*th position within the class will be different from the unconditional distribution of nucleotides at the same position over the complete set of tRNAs. It is possible to estimate corresponding distribution dissimilarity (AKA distribution divergence, distance); furthermore, we assume that the higher the dissimilarity, the more likely is the associated position to play a significant biological role for the specific subgroup of tRNAs considered. Otherwise, we assume the position to be relatively “unimportant” for this tRNA subgroup—although it might still have an overall functional significance, for all tRNA molecules in general.

Formally, given the probability space (ω,⊣,μ) and a set of categorical random variables with range defined by the set of nucleotides and a gap symbol, s=(a,c,g,u,_)

$$x_{i}:\omega \to s,$$

we denote the distribution at *i*th position of the alignment by

(1) $$q_{i}=\mu \left(x_{i}\right).$$

**Figure 3:**
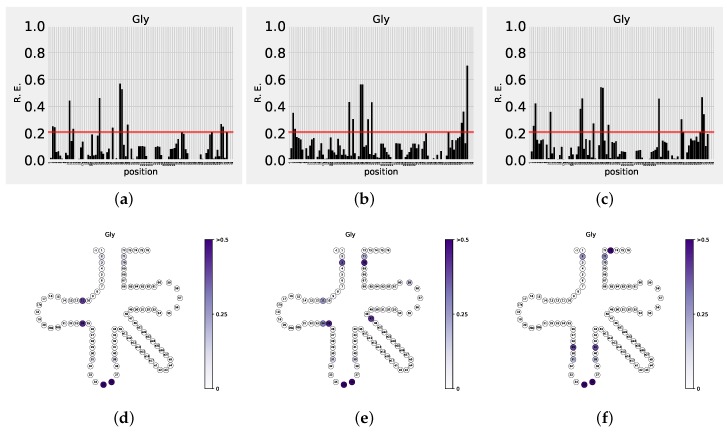
Position “importance” profile for Gly tRNAs, shown for three life domains: Archaea (**a**,**d**), Bacteria (**b**,**e**) and Eukarya (**c**,**f**). Relative Entropy is shown as function of tRNA position (**a**–**c**) (enumerated as in Figure 1, first row), or visualized as color intensity superimposed over the secondary tRNA structure (**d**–**f**). Significance cutoff limit is shown as a red line in (**a**–**c**)—see text for discussion.

**Figure 4:**
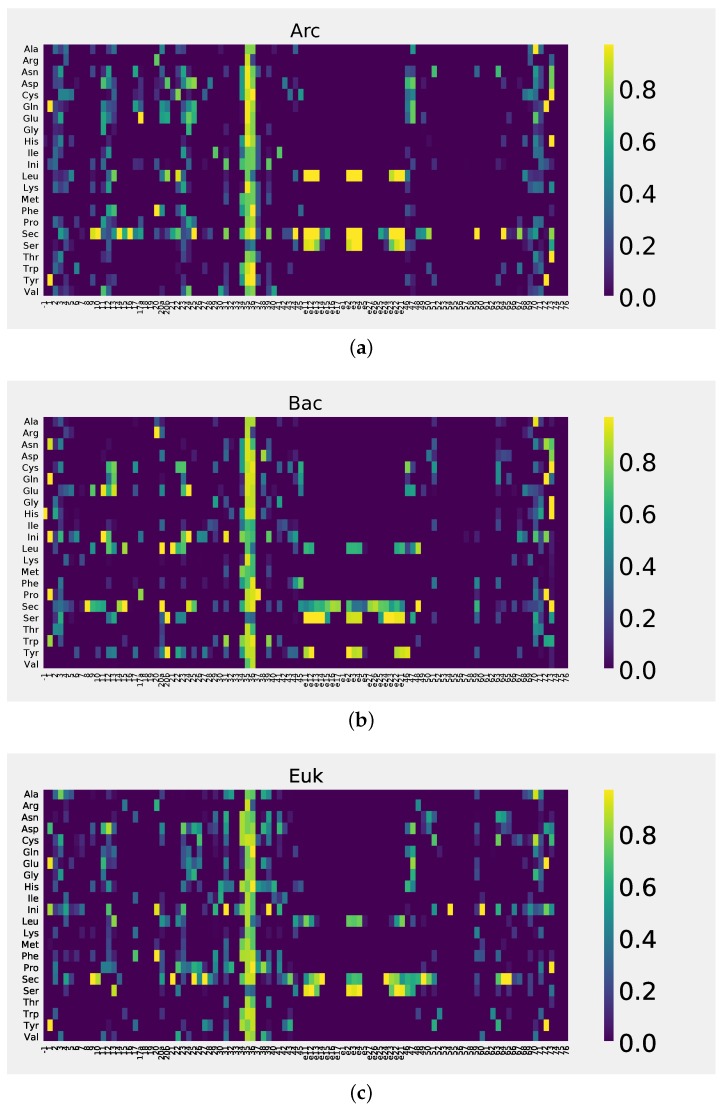
Summary visualization of the tRNA position “importance”, for all aa tRNA subclasses, shown for three life domains: Archaea (**a**); Bacteria (**b**) and Eukarya (**c**). Higher values correspond to “hotter” colors. tRNA position numbering is as in Figure 3. This is a summary visualization of the detailed plots presented in Supplemental Figures S1–S22.

Note that the gap state here is used for convenience in interpreting the results in Figure 3 and Figure 4 (and Supplemental Figures) only. It was not used in the BN construction/interpretation. Instead, during the BN reconstruction, dependencies between positions 17a, 20ab, e1-27 were treated as (comparatively) rare events, under multinomial local probability models in correspondingly reduced datasets.

Given the categorical random variable *y* with the same sample space ω and range *a* defined by the set of amino acid classes $a=\left\{a_{j}\right\},$

y:ω→a,

we define the conditional distribution of nucleotides at each position *i* for a given class of amino acids $a_{j}$ as

(2) $$p_{ij}=\mu \left(x_{i}\cap \left[y=a_{j}\right]\right).$$

Our goal is then to estimate the dissimilarity between $p_{ij}$ and $q_{i}$ using some appropriate dissimilarity or distance measure. For this purpose, we select the (symmetrised and smoothed) measure of relative entropy *l*; its square root is a proper metric (distance function), which is a desirable property. We define

(3) $$l\left(p_{ij},q_{i}\right)=\frac{1}{2}d\left(p_{ij},m_{ij}\right)+\frac{1}{2}d\left(q_{i},m_{ij}\right),$$

where the operator d(x,y) is

(4) $$d\left(x,y\right)=\sum_{k}x\left(k\right)\log\left(\frac{x(k)}{y(k)}\right)=h\left(x,y\right)-h\left(x\right),$$

and

(5) $$m_{ij}=\frac{1}{2}\left(p_{ij}+q_{i}\right).$$

(Below, we will occasionally refer to $l(p_{ij},q_{i})$ as simply $l_{ij}$, where appropriate.) The quantity denoted by $l_{ij}$ is the symmetrised measure of relative entropy (RE), also known as Jensen–Shannon divergence, or JSD ([21,22]; conveniently, its square root defines a mathematically correct distance function. Because the image of *l* is always a finite interval im(l)=[0,max(l)], the results (inter-distributional differences) are easy to interpret—when *l* is close to 0, the distributions that are being compared are very similar, whereas, when *l* is close to its maximum value, the distributions have little to nothing in common. Conveniently, this allows us to superimpose an absolute scale on the degree of “importance” that a particular position has within the context of a given class of tRNAs—not only can we contrast the relative importances between different positions, but we can also estimate how important any given position is in absolute terms.

A series of plots (Figure 3 for Gly, the simplest, most basic, amino acid, example; Supplemental Figures S1–S22 for all amino acids) shows the “importance” profile for each class of tRNA in *y*, partitioned into the three life domains, Archaea (Arc), Bacteria (Bac), and Eukarya (Euk). A brief glance at the charts reveals that each tRNA class possesses a unique “fingerprint” pattern of RE values assigned to the tRNA positions. Throughout all the classes, however, certain general features are easily identifiable: for instance, codon position (34, 35, 36) shows high “importance” in all tRNA groups; on the other hand, invariant and semi-invariant positions are associated with low “importance” (see Figure 4 for a heat plot summary visualization of tRNA position importance, by life domain (Figure 4a–c), tRNA class, and nucleotide position). The next obvious question is: just how high is sufficiently (significantly) high? Obviously, positions 35, 36 are significant, and, for example, position 33 is not. What about position 34? Throughout most of this study (see also Table 1 below), we have set a somewhat arbitrary significance cutoff value of RE, at 0.2 (marked by the red horizontal lines in Figure 3 and Supplemental Figures S1–S22), largely following current literature and visual analysis of the distributions. In order to impose a rigorous quantitative cutoff measure, however, we can generate a set of random samples $y_{j}^{*}$ from *y* to obtain

$$p_{ij}^{*}=\mu \left(x_{i}\cap y_{j}^{*}\right).$$

This operation is expected to have the effect of scrambling any probabilistic relationship between *y* and $x_{i}$ (unless there is a systematic bias in the data). We then define

$$l_{ij}^{*}=l\left(p_{ij}^{*},q_{i}\right)$$

and obtain a sequence ${\left\{l_{ij}^{*}\right\}}_{j=1}^{m}$ induced by the random sampling of *y*, which should correspond to the distribution $h_{0}$ of random noise in the data. In our experiments, setting *m* at $10^{5}$ was sufficient for convergence. Whenever the observed value of $l_{ij}$ exceeds this “random noise” threshold, it is reasonable to suspect that there is a non-trivial signal. It appears that, for all amino acid classes, in almost all positions, this is indeed the case. Therefore, it is highly improbable that most observed $l_{ij}$ values could have resulted from the random processes. To put it bluntly, almost nothing about most tRNA positions is even close to random.

Of course, this is not at all unexpected from the purely biological viewpoint—this observation is simply a confirmation of the fact that the tRNAs for a certain amino acid class are not independent and that they share specific structural and sequence information.

What was not expected, however, is that, at almost every position, the distribution between the tRNA classes is highly non-random. Two factors likely attribute to this effect: first, the vestiges of the phylogenetic relationship between the tRNAs; and, second, structural and functional aspects associated with each tRNA class. After having observed the non-random signal associated with a particular tRNA position (as detailed in Supplemental Figures S1–S22 and summarized in Figure 4), we now need to establish whether it is not only “high”, but also “specific” (to the tRNA class). The non-specific signal can be attributed to some other kind of underlying relationship (e.g., a structural one); in general, such signal is a second- (or higher-) order relationship compared to the tRNA class-driven one. It can also be interpreted as a “background” signal, present irrespective of the tRNA class stratification. It is intrinsic to the tRNA structure in general but has little to no relevance regarding amino acid specificity. These higher-order signals are unlikely to be directly responsible for any specific tRNA function but will be strong enough to show as non-random effects. In this context, we posit that the positions with “high” and “specific” signals are the ones with the highest likelihood of being “important” (for the biological function of a particular tRNA class). They are preserved within, and discriminate between, specific tRNA groups. In contrast, in “non-specific” positions, non-random signal simply either reflects the echoes of the phylogenetic relationships, not yet completely dissolved, or points to the general tRNA structural relationship patterns.

In summary, by selecting the positions with “high” and “specific” RE signal, we zero in on the positions crucial for the biological functions and features of specific tRNA groups (as opposed to the “background”, or higher-order, signal in non-specific positions associated with the phylogenetic, structural, etc., aspects of all the tRNAs).

One way to separate the latter (not directly related to the amino acid specificity) positions would be to compute RE after conditioning the data for the states in position *i*, and repeating for all *n* positions, thus obtaining a new collection of distributions:

(6) $$z_{iqr}=\mu \left(x_{q}\cap \left\{x_{i}=r\right\}\right),$$

where *r* is one of the possible states (nucleotides/gap) at the position *i* with position q≠i. By using this procedure, one can establish the baseline RE distribution associated with each position.

### 3.3. Identification, in the Three Domains of Life, of the tRNA Amino Acid Specificity Identity Determinants

Typically, in the literature, the notion “tRNA identity determinants” refers to the nucleotide positions that are recognized by aaRSes, and are involved in the loading of tRNAs. Here, we expand on that definition. By “identity determinants” we mean that all of the nucleotide positions that are potentially predictive with respect to identifying a particular tRNA subclass. Table 1 summarizes the quantitative results of our analyses (by a given amino acid, and the three domains of life).

Here, we will discuss Gly tRNA as an example (see also Figure 3 a–c for the exact RE values). The rationale between this choice is two-fold: first, Gly is obviously the simplest possible, most basic, amino acid. Second, it is well-represented in the tRNA database, thus making this example particularly robust. Consider bacterial Gly tRNAs (Figure 3b). Immediately, positions 2-71 and 3-70 are confirmed as important ones for the tRNA identity. However, positions 10-25 and 1-72, previously recognized by means of experimental analyses [2,25], do not appear to be identity determinants in our analysis. Interestingly, 1-72, 2-71 and 3-70 are all present in the same ancestor stem; while the experimental analysis supports all three, our information theory-based analysis implies that the 1-72 pair is not really involved in determining the identity of Gly tRNAs in the Bacteria domain. Is the experimental analysis pinpointing 1-72 simply incorrect, and thus should be further validated? A similar scenario unfolds in the case of positions 10-25, supported by the experimental analysis; instead, our analysis suggests 29-41 and 31-39. These latter pairs apparently constitute the kind of identity that might not involve the aaRSes at all. Moving on to Eukarya (Figure 3c), 2-71 and 3-70 are confirmed by our analysis as well; 1-72 and 10-25 are not supported by either experimental or information-theoretic analyses. Neither are they supported by our analysis in Archaea Gly tRNAs (Figure 3a). This seems to strengthen the suggestion that 1-72 and 10-25 pairs are not in fact involved in determining the identity of Gly tRNAs in all domains, in spite of the experimental (aaRSes) evidence. On the contrary, positions 31-39 (in addition to 2-71 and 3-70) are supported by our analysis for all three domains. This hints at a strong signal because it is persistently present in smaller, relatively independent (or at least sufficiently heterogeneous) tRNA subsets.

Another representative example is the Ala tRNAs, where our analysis confirmed all known critical position (e.g., position 3-70 for Bacteria and Eukarya). For Ala tRNA, our analysis further suggests potentially important positions, namely 31-39 in the anticodon stem and 32,38 in the anticodon loop for Eukarya, and 20a for Archaea, Bacteria and Eukarya. Such analyses can of course be extended to all amino acid identity determinants, dovetailing with the existing and ever-growing literature. This largely statistical approach should ideally precede (or augment) the experimental work, in suggesting the possible (and likely) identity determinants to be pursued and validated experimentally. For instance, just by looking at Figure 3, positions 31-39 would be a strong candidate in the case of Gly tRNAs. Detailing chemistry and biochemistry of particular tRNAs and their positions is outside the scope of this manuscript—rather, it is a general future research direction for both ourselves and investigators elsewhere, armed with the methodology presented in this communication. (Specifically, we intend to follow up with a comprehensive, more detailed, analysis of the tRNA families for which both phylogenetic/alignment data and atomic structures of their complexes with aaRSes are available.)

## 4. Discussion and Future Directions

While probabilistic directed acyclic graph (PDAG) representation (as realized in the BN) is a conveniently compressed, informative and intuitively appealing visualization of the probabilistic relationships observable in the data, when it comes to fine detail scrutiny, high order abstraction can be a shortcoming. The information contained in conditional node distribution is difficult to summarize and interpret in its totality because of the multitude of all possible states. For instance, for a node with the three edges, one would have to scrutinize a 27x3 conditional probability table only to get a partial picture. In addition, it would be a partial picture because formation of the comprehensive conditional probability table would require inference over the complete Markov blanket of the node (which, in most cases, would include more than the immediate neighbor nodes) (see [18,19] for a comprehensive discussion of conditional independence and “Markov blankets” in directed graphs).

Recall that one of the motivations of our research was to study amino acid group-specific changes in tRNAs, or, to paraphrase, to develop a procedure for (1) conditioning on the aa variable; and (2) somehow comparing, in a quantifiable way, the resulting group-specific dependence structures. Building BNs for each and every aa class is one such procedure; however, quantifying topological differences between resulting networks is a difficult problem. Indeed, it is not enough to just “count” the presence or absence of a specific edge in the network—one would have to establish which particular set of differences pushes the model outside of the BN equivalence class [18,19]. While doable, this necessitates further BN methodological development, which is outside the scope of this paper (but is a research direction we are pursuing presently). Of course, there is also an issue of decreased sample sizes (when stratifying by aa variable). *(We remind here that all of the BN built for the different tRNA subclasses (stratified by aa and three life domains) are available directly from the authors.)*

An alternative way to deal with the complexity of the situation (i.e., “top-down” analysis from the high order abstraction of the BN topology to the individual tRNA determinants), described in detail in the previous section, required making two crucial simplifications. First, we restrict further (“downstream”) analysis to pairwise relationships (conditioned on the aa class variable) only. This step compresses much of the dynamics into one low order approximation for every group that can be easily assessed and compared by means of simple charts (as in Figure 3 and Supplemental Figures S1–S22) and 2D color arrays (as in Figure 4). Second, we switch from the more comprehensive model scoring function (the MDL principle that underlies our BN structure learning algorithm) to a Mutual Information (MI)-based criterion that retains the desired feature of information theoretic entropy in order to condense the values of a given multidimensional probability distribution to a single distance-like value (namely, RE).

In summary, our procedural approach is a three-pronged one:

(1) Build the BNs for the tRNA sub- and super-classes of interest. Visually compare and contrast topologies. Interpret the intra-tRNA features (dependencies between the positions, pairwise and more complex) and inter-tRNA class differences.
(2) In parallel, use measures of distribution divergence to identify aa class-specific tRNA identity positions.
(3) For such positions, go back to the results of (1) to identify and interpret dependencies between these positions and other tRNA sites.

Ideally, this process can be further automated by developing routines for directly querying and performing statistical inference within Markov blankets (conditional probability tables) of nodes of interest, and for direct comparison of BN topologies by differentiating the equivalence classes associated with them. This is an ongoing research direction.

Our approach, as implemented in this study, is conceptually similar to the recent data-driven analyses of tRNA databases [26,27]. Galili and colleagues used a decision-tree based classifier to perform a variable selection (embedded in classification) to derive the *minimal* set of tRNA identity/informative positions for each amino acid in Arc domain. This is also a machine learning-based approach; decision tree-based classifiers are in many ways a complementary technique to the BN modeling. However, single decision trees (such as Classification and Regression Trees (CART) used in the analysis) are notorious for getting stuck into the local extremums, especially at the earlier stages of decision process (corresponding to the more important tRNA positions, in our case). Therefore, generated tRNA identity/informative position sets are, while undeniably compact, potentially biased, and might not be particularly robust. This is especially true for smaller datasets with more “difficult” dimensionality (such as aa-specific tRNA subclasses). Ideally, such sets should be at least complemented by the variable sets generated via methods espousing more exhaustive search (BNs, or ensemble decision tree-based classifiers). Depending on the extent of decision tree pruning, single decision tree-based classifiers can also be relatively blind to the rare variants (which is not a drawback that BNs share). In our opinion, it would be a very fruitful research direction to adapt the analyses in [26] to more robust ensemble classifiers, such as Random Forests with double-loop cross-validation [28]. At this time, however, we believe that the robustness and exhaustiveness of the variable sets generated by our approach is preferable to the compactness of the sets generated by a single decision tree—especially in the context of the exploratory, hypothesis-generating analysis activity. In statistical terms, the latter is simply too false negative-prone. Galili et al. [26] clearly recognize the shortcomings of such “greedy” algorithms as CART; however, they conclude that combinatorial efficiency and compactness of the results outweigh such drawbacks. We will re-emphasize here that CART and BNs, in this context (of predominantly exploratory analytic activity), complement (rather than compete with) each other.

Zamudio and José ([27]) used mutual entropy to discover isoacceptor tRNA positions that form “clusters” with respective anticodons. (This is mathematically similar to SNP linkage disequilibrium—except, of course, that the underlying biology, whatever it might be, is likely different in most cases.) In statistical terms, this presents a univariate testing alternative to our analysis (which is multivariate by design), with all the intrinsic advantages and disadvantages thereof. The primary advantage is that it allows to “manually” zero in on the primary intra-tRNA relationships of interests (such as identifying tRNA isoacceptor sites that are highly related to anticodon). Consequently, the primary disadvantage is the inability to capture more complex (than pairwise), non-additive, relationships. Similarly, although pairwise testing is obviously very computationally efficient, as a strategy it is essentially a series of independent tests that has to be carried out “manually”, which is not conducive to the automated hypothesis generation (a hallmark of machine learning methods such as BNs and, to a somewhat lesser degree, decision tree-based classifiers). In our view, BN analysis is the method of choice for automatically identifying (and visualizing) potentially interesting intra-tRNA relationships. These can be further scrutinized using univariate testing such as developed by Zamudio and José [27], or conditional probability tables associated with the specific nodes/edges in the BN context.

Our approach can also be highly useful in addressing the specific problem of predicting tertiary (3D) tRNA interactions. Briefly, the problem can be defined as follows: thorough analysis of tRNA sequences (such as carried out in this study, and by us and others before) can be used to derive the basic secondary (2D) structure of tRNA. Standard 2D structure prediction algorithms aim to find thermodynamically stable 2D tRNA structures that minimize free energy given the primary tRNA sequence. This is a straightforward search/optimization task, and it is largely tractable within the tRNA sequences domain, assuming standard complementary base pairings hold (see [29] for a recent example). The resulting 2D structure is represented by a familiar tRNA “cloverleaf” consisting of the helix (stem) and the loop regions—the former subject to canonical Watson–Crick and GU pairing rules—the latter, less constrained. We now move on to the prediction of 3D tRNA structures (also known as “l-shape”), and this is a much more daunting task. Part of the intrinsic difficulty is that 3D structure prediction usually relies on the 2D structure (as a starting point), to which thermodynamics- and kinetics-driven operators (reflecting relatively minor free energy changes) are subsequently applied. This, of course, suffers greatly from the cumulative “dead reckoning” effect. Another complication is that 3D tRNA structures do not exist in a vacuum, but are shaped in the process of interacting with other molecules within different cellular environments (mitochondria, ribosomes, etc.). In addition, yet another (related) complication has to do with the formal definition of operators (reflecting energy changes in 2D/3D structure search/optimization process). They are based on tabulated thermodynamic parameters estimated under “standard” fixed conditions (temperature, pH, ionic strength), which might or might not hold under different circumstances (varying cellular environments, interacting proteins and other RNAs, etc.).

The work of Mustoe et al. [30] is a representative recent example of in silico 3D tRNA analysis that had to be complemented by experimental work in order to reach unequivocal conclusions. Here, just as with the more specific tRNA – aaRS coupling problem, experimental methods become essential. Expensive and time-consuming procedures (crystallographic and spectroscopic techniques) can obtain, or confirm, structural information, but even then there is no guarantee that the results generalize to in vivo. In particular, there is usually a number of alternative, however similar, optimal structures, and the one that reaches the absolute minimum (in free energy) in silico or in vitro is not necessarily the one actually functioning in vivo.

Thus, we return to the notion of the automated data-driven discovery of “interesting” polymorphisms/structural features/interactions within the tRNA molecules that would otherwise be difficult to capture because of an altogether unknown biological context, or prohibitively high predictive algorithm complexity, or modeling assumption violations, or simply sheer time and expense of experimental work.

However, another benefit of the tRNA BN analysis lies in its potential utility for tRNA phylogenetic reconstruction. Applying standard nucleotide distance metrics, or nucleotide transition probabilities (usually a cornerstone of phylogenetic reconstruction) to the tRNA molecules is questionable for a number of reasons ([31] for recent discussion). However, information contained in structural (topological) differences between BNs estimated for specific subclasses of tRNAs can be used to measure phylogenetic distances between these subclasses, which can be further translated into the phylogenetic hierarchy. In fact, unlike with some ad hoc metrics, differences in BNs by their very nature capture the “holistic” (more complete, comprehensive) picture of the differences between a given pair of subclasses—indeed, the sole purpose of BNs is to reflect the significant probabilistic relationships contained in the “flat” data, regardless of the underlying nature of these relationships. Assuming that these observed probabilistic relationships reflect true biological interactions (and not random noise)—a reasonable assumption for the sufficiently large datasets, such as the currently available tRNA data—they should be a viable foundation for the analysis of tRNA gene histories.

Such BN-based approach to tRNA phylogeny is conceptually similar to, and would complement, the approach espoused in [31], which prioritizes “anchor elements” based on syntenic tRNA properties as the most useful for deriving phylogenetic information. The rationale behind the latter strategy is that relatively stable synteny anchors are less susceptible to the randomizing and obfuscating effects of the rapid-churn concerted evolution of gene families (such as tRNAs). Similarly, topological differences between BNs built from the different tRNA subclasses are less sensitive to the evolutionary (background) noise, and reflect robust features that have been/are being fixated during the correspondent tRNA subclasses’ formation. One of the intriguing future directions of our work is to trace tRNA gene histories using BN topological distances and see how the results compare to the synteny-based phylogenies of tRNAs in, for example, fruit flies and primates [31].

## 5. Conclusions

If there is one thing that can be said with certainty about the tRNA world, both extant and extinct, is that it is/was arguably much more complex (quantitatively and qualitatively) than what we used to think as recently as two to three decades ago ([32] and references therein). Amusingly, it has been suggested [32] that the ongoing evolution of the multitude of tRNA species can best be described by analogy with an artificial intelligence system (specifically, an interplay of minimax algorithms). This might be a stretch; if anything, tRNA populations can be better approximated by the “genetic algorithms” (which, in turn, were developed to mimic natural genetic/evolution processes in a first place). However, whimsicality aside, we do believe that artificial intelligence methodology (specifically, machine learning techniques) is the best way to approach secondary data analysis of growing tRNA sequence databases, be it for the purposes of tRNA structural research, identity elements cataloging, unraveling tRNA-disease associations, or tRNA evolutionary studies.

This brings us to the problem of genetic code and translation machinery origins, a subject unequivocally linked with the tRNAs and their (early) evolution. A recent review by Koonin and Novozhilov ([33]) suggests, among other things, that “theoretical study of the genetic code as a cryptographic problem has largely run its course”. In our opinion, this sentiment, while having a defeatist flair about it, is fundamentally correct. The consensus understanding, at this time, revolves around the “synthesis” of stereochemical affinity hypothesis, and frozen accidents alongside universal genetic code code expansion. The former largely hinges on the "important" tRNA sites other than the anticodon, automated discovery of which is of course the primary goal of this study. This should be followed by the experimental work as well as dynamical modeling, along the lines suggested by Koonin and Novozhilov [33], Carter and Wills [34], Martinez-Rodriguez et al. ([6]), to name just a few.

Such experimental work is time-consuming and costly; not coincidentally, one of the primary deliverables of this study is the list of "suggested" tRNA positions of interest (third column in Table 1). Moreover, it is possible to "rank" the suggested positions using the RE metric. For any specific subgroup of tRNAs, investigators can identify the suggested (and so far experimentally unverified) tRNA positions and simultaneously rank/select them using RE and/or various RE cutoff criteria. The resulting selected positions can then be scrutinized by identifying other tRNA positions associated with them in the appropriate tRNA subgroup BNs. After subsequently evaluating (and further prioritizing) these selected positions from the evolutionary, biochemical, biological and structural (2D, 3D) viewpoints, one can proceed to the thus more precisely guided experimental work.

On a philosophical note, even unequivocal experimental validation of the processes suspected to have taken place during the genetic code/translation apparatus origin does not yet prove that these events actually took place. There are many possible, and perfectly realistic, alternative pathways leading to the extant universal genetic code; the sheer volume of related literature (and hypotheses and scenarios suggested therein) attests to the fact. Experimentally reproducing one of them does not preclude the possibility of another, and does not necessarily bring us that much closer to the definitive proof of “what has really happened”. Similarly, for in silico work, the Occam’s razor approach (which underlies much of the machine learning research by directing us to seek the most compact system/model fitting the observed data the best) is subject to a parallel logical fallacy, that of assuming that nature (and evolution) deals with globally optimized, at any given moment, biological systems (“greedy search” of locally optimized states being significantly closer to the reality of evolutionary processes). At any rate, while the purely “cryptographic” approach to the elucidation of universal genetic code origins might feel unsatisfying [33], we strongly believe that non-trivial relationships and associations observed in the extant molecules (principally tRNAs and aaRSes) can and should point to the more likely (statistically and experimentally) scenarios of code origin, or at least to certain likely evolutionary steps along the way. Much of the recent work in the field has been pursued following this paradigm [27,35,36,37].

Detailing plausible scenarios of genetic code origin is outside the scope of this study; rather, our goal here was to present a useful instrument and methodological framework for identifying statistically significant relationships between the positions within the tRNA molecule and between different subclasses of tRNAs. It is our intention for this novel methodology to assist the investigators in pinpointing non-random patterns of change in tRNAs, at both micro- and macro-evolutionary levels.

## Figures and Tables

**Table 1:** Determinant tRNA positions classified by the amino acid and the three domains of life (Archaea, Arc; Bacteria, Bac; Eukarya, Euk). Only positions with the Relative Entropy (RE) value higher than the preset cutoff value (set at RE approx. 0.2, as illustrated in Figure 3 and Supplemental Figures S1–S22) are shown. Note that the preset cutoff value is higher than that suggested by the “random noise” ${\left\{l_{ij}^{*}\right\}}_{j=1}^{m}$ sequence (see text). For compatibility with the literature, the position numbering system follows that of [23,24]. It corresponds to the first row in Figure 1. Paired bases in the secondary structure of tRNA are shown as connected by a dash; for example, the 3-70 pair indicates that in the secondary structure of the tRNA molecule, the bases at position 3 and 70 should be paired [23,24]. Similarly, “=” indicates tertiary interactions among tRNA nucleotides. Question marks indicates the uncertain positions, that is to say positions that might not be important for the tRNA molecule. “Confirmed” indicates that the nucleotides identified as “important”, or identity determinants, in the experimental analyses [2,25] were also significant (high RE values) in our analysis. “Low significance” means that the “experimentally proven to be important/identity determinants” nucleotides were not associated with high RE values. On the contrary, “Suggested” positions exhibit high RE values but so far have not been registered in the experimental analysis literature.

Amino Acid	Confirmed	Low Significance	Suggested	Domain
			2-71; 3-70; 4-69; 9; 12-23; 13-22	Arc
			20a; 30-40; 35; 36; 44; 47	
Ala	2-71; 3-70; 4-69; 20; 64; 73		17; 20a; 29-41; 35; 36; 44; 51-63	Bac
	3-70		2-71; 4-69; 5-68; 9; 12-23; 13-22; 20a; 27-43	Euk
			29-41; 31-39; 32; 35; 36; 38; 59	
			4-69; 20; 35; 36; 73	Arc
Arg	20; 20A;	38; 73	4-69; 5-68; 20a; 35; 36	Bac
			4-69; 15; 20; 35; 36; 48; 71	Euk
			2-71; 3-70; 9; 11-24; 12-23; 17; 17a	Arc
			20a; 22; 31-39; 34; 35; 36; 37	
			46; 47; 51-63; 59; 73	
Asn	73		1-72; 2-71; 3-70; 12-23; 31-39	Bac
			32; 34; 35; 36; 51-63	
			2-71; 4-69; 13-22; 17; 27-43; 29-41	Euk
			31-39; 34; 35; 36; 38; 49-65	
			50-64; 51-63; 59; 73	
			2-71; 3-70; 6-67; 11-24; 12-23; 13-22; 17a	Arc
			20b; 20a; 20; 25; 28-42; 34; 35	
			36; 44; 46; 47; 49-65; 64; 73	
Asp	25; 38; 73	2-71; 10	11-24; 20a; 31-39; 34; 35; 36	Bac
			43; 44; 50-64; 51-63; 65	
	9-12-23; 25; 38; 73	10	1-72; 11-24; 13-22; 20a; 26; 28-42	Euk
			29-41; 31-39; 34; 35; 36; 46	
			47; 49-65; 50-64; 59; 63; 71	
			3-70; 4-69; 5-68; 12-23; 13-22; 17	Arc
			17a; 20; 21; 24; 27-43; 34	
			35; 36; 45; 46; 47; 73	
Cys	2-71; 3-70; 13-22; 46; 73	15; 48	12-23; 17; 29-41; 34; 35; 36	Bac
			43; 45; 47; 51-63; 71	
	12-23; 73	20a	2-71; 3-70; 6-67; 7-66; 9; 13-22;	Euk
			26; 29-41; 31-39; 34; 35; 36; 37	
			38; 51-63; 59; 68; 69	
			1-72; 2-71; 3-70; 4-69; 11-24; 12-23; 13-22	Arc
			17; 17a; 20b; 20a; 25; 34; 35	
			36; 37; 44; 46; 47; 73	
Gln	1-72; 38; 73	2-71; 3-70; 10-25; 37	12-23; 13-22; 20a; 34; 35; 36	Bac
			44; 45; 46; 51-63; 65	
			2-71; 3-70; 6-67; 7-66; 11-24; 12-23	Euk
			13-22; 26; 29-41; 31-39; 34; 35	
			36; 44; 46; 47; 52-62; 73	
			2-71; 3-70; 11-24; 12-23; 13-22; 17a; 20a; 20b	Arc
			25; 34; 35; 36; 46; 47; 49-65	
Glu	11-24; 13; 46; 47; 71	1-72; 22; 33; 37	3-70; 4-69; 5-68; 7-66; 9; 12-23	Bac
			17; 20a; 30-40; 34; 35; 36	
			38; 45; 49-65; 51-63	
			1-72; 2-71; 3-70; 5-68; 11-24; 12-23	Euk
			13-22; 25; 26; 31-39; 34; 35	
			36; 38; 47; 59	
			2-71; 3-70; 11-24; 13-22	Arc
			31-39; 35; 36; 49-65	
Gly	2-71; 3-70; 73	1-72; 10-25	29-41; 31-39; 35; 36; 63	Bac
	2-71; 3-70	73	11-24; 25; 31-39; 35; 36; 47; 59	Euk
			-1; 2-71; 3-70; 5-68; 11-24; 12-23	Arc
			34; 35; 36; 37; 50-64; 73	
His	-1; 73		2-71; 3-70; 4-69; 6-67; 31-39	Bac
			32; 34; 35; 36; 38; 63	
		-1; 73	2-71; 9; 11-24; 12-23; 13-22; 26	Euk
			30-40; 31-39; 32; 34; 35; 36	
			37; 38; 44; 45; 46; 47	
			2-71; 3-70; 11-24; 12-23; 29-41; 31-39	Arc
			34; 35; 36; 37; 46; 73	
Ile	12-23; 29-41	4-69; 24; 37; 38; 73	3-70; 6-67; 13-22; 20a; 27-43; 28-42	Bac
			34; 35; 36; 44; 51-63	
			4-69; 17; 20a; 28-42; 29-41	Euk
			30-40; 34; 35; 36; 60	
			1-72; 2-71; 9; 11-24; 17; 17a; 20	Arc
			20b; 27-43; 31-39; 34; 35; 36; 37	
			47; 51-63; 57; 64; 73	
Ini	2-71; 3-70; 32	33; 37	1-72; 5-68; 6-67; 11-24; 12-23; 17a	Bac
			26; 27-43; 29-41; 31-39; 34; 35	
			36; 44; 57; 59; 73	
			1-72; 2-71; 3-70; 4-69; 5-68; 6-67; 7-66	Euk
			12-23; 20; 20a; 22; 27-43; 29-41; 31-39	
			33; 34; 35; 36; 38; 46; 51-63	
			54; 59; 60; 73	
			2-71; 3-70; 4-69; 5-68; 9; e11	Arc
			12-23; 13-22; 20a; 20b; e21; 31-39	
			35; 36; 37; 44; ; 46	
Leu	20A; 73	20; 38;	2-71; e5; 9; e11; 12-23; 13-22; 15; 20a; 21	Bac
			e21; 35; 36; 44; ; 46; 47; 48; 73	
			4-69; e5; e11; 12-23; 13-22; 20a	Euk
			20b; e21; 29-41; 35; 36; 37	
			44; ; 45; 47; 49-65; 68	
			2-71; 3-70; 4-69; 9; 11-24; 12-23; 22	Arc
			31-39; 34; 35; 36; 37; 46; 73	
Lys	73?		4-69; 5-68; 7-66; 12-23; 20a; 26	Bac
			31-39; 34; 35; 36; 73	
			2-71; 7-66; 9; 12-23; 13-22; 17; 20a; 29-41	Euk
			31-39; 34; 35; 36; 44; 59; 70	
			31-39; 34; 35; 36; 37; 73	Arc
Met	73	4-69; 5-68; 38	31-39; 34; 35; 36; 71	Bac
	20	73	1-72; 12-23; 31-39; 34; 35; 36; 60; 64	Euk
			9; 12-23; 13-22; 20; 20a; 34; 35	Arc
			36; 37; 45; 46; 47; 73	
Phe	27-43; 31-39; 44; 45; 59	20?; 28-42; 30-40; 37; 39?; 43?; 60; 73?	3-70; 12-23; 17; 20a; 34; 35	Bac
			36; 39; 43; 51-63; 73	
	20; 31-39; 37	73?	2-71; 4-69; 5-68; 6-67; 9; 12-23	Euk
			13-22; 17; 20a; 29-41; 34; 35	
			36; 51-63; 59; 60; 73	
			2-71; 3-70; 6-67; 11-24; 12-23; 13-22	Arc
			17a; 25; 35; 36; 37; 46	
Pro	72; 73	15; 48	1-72; 2-71; 3-70; 17a; 35	Bac
			36; 37; 44; 59	
			2-71; 11-24; 12-23; 13-22; 20a; 25	Euk
			26; 27-43; 29-41; 31-39; 32; 35	
			36; 37; 38; 49-65; 73	
			3-70; 4-69; 7-66; 9; 10-25; e11; 11-24; 12-23	Arc
			13-22; 14; 15; 16; 17a; 17; 20b; 20	
			20a; 21; e21; 27-43; 28-42; 31-39; 34; 35	
			36; 37; 44; ; 48; 49-65; 50-64; 59	
			67; 68; 72; 73	
Sec	2-71; 3-70; 4-69; 5-68; e5; 7-66	1-72; e2; e3; e4; 6-67; 12-23; e12; e13	14; 15; 16; 20a; 29-41; 31-39; 34; 35	Bac
	8; 9; 10-25; 11-24; e11; e17	13-22; 20; e22; e23	36; ; 59; 63; 64; 66; 68	
	e21; e27; 45; 48; 73; e24; e25			
	e26; 50-64; 64?; 66?; 68?		
			2-71; 4-69; 5-68; 9; 10-25; e11; 14; 20a	Euk
			e21; 21; 23; 26; 27-43; 28-42; 29-41; 31-39	
			34; 35; 36; 38; 44; ; 46; 47	
			48; 49-65; 50-64; 51-63; 59; 66; 67; 73	
			2-71; 4-69; 5-68; e11; 12-23; e21; 22; 24	Arc
			35; 36; 44; ; 46; 47; 73	
Ser	2-71; 3-70; e4-69;11; e21; 44; 73	11-24	5-68; 12-23; 13-22; 20a; 20b; 35	Bac
	e4; e5?;e12; e13; e14; e15; e16; e22		36; ; 46; 47; 51-63; 59	
	e23; e24; e25; e26; 69? e2; e3;			
	e11; e21; e2; e3; e4; e12; e13; e22; e23		4-69; 13-22; 20a; 23; 27-43; 35; 36; 44	Euk
			; 46; 47; 49-65; 51-63; 59; 73	
			2-71; 3-70; 11-24; 12-23; 35	Arc
			36; 37; 46; 73	
Thr	2-71; 3-70	1-72; 73	20a; 35; 36	Bac
		1-72	31-39; 35; 36; 73	Euk
			2-71; 3-70; 6-67; 22; 27-43	Arc
			31-39; 34; 35; 36; 50-64	
Trp	1-72; 3-70; 73	2-71; 5-68; 9	15; 20a; 29-41; 31-39; 34; 35; 36; 48	Bac
			2-71; 15; 20a; 31-39; 34; 35	Euk
			36; 43; 48; 52-62; 65	
			1-72; 4-69; 9; 12-23; 13-22; 31-39; 34	Arc
			35; 36; 37; 46; 47; 51-63; 73	
Tyr	73		e5; 6-67; 10-25; e11; 12-23; 13-22; 17; 20	Bac
			20a; 20b; e21; 27-43; 28-42; 31-39; 34; 35	
			36; 44; ; 46; 59; 71	
	1-72	73	12-23; 17; 27-43; 28-42; 31-39	Euk
			34; 35; 36; 51-63; 70	
			2-71; 3-70; 4-69; 5-68; 6-67; 11-24; 12-23	Arc
			20a; 30-40; 31-39; 35; 36; 47	
Val	73	3-70; 4-69	13-22; 35; 36	Bac
			3-70; 11-24; 12-23; 13-22; 27-43	Euk
			31-39; 35; 36; 38; 60	

## Supplementary Materials

The following are available online at http://www.mdpi.com/2075-1729/8/1/5/s1, Figures S1–S22: Position “importance” profile for all aa tRNA subclasses, shown for three life domains, Archaea (**a**,**b**), Bacteria (**c**,**d**) and Eukarya (**e**,**f**). Relative Entropy is shown as function of tRNA position (**a**,**c**,**e**) (enumerated as in Figure 1, first row), or visualized as color intensity superimposed over the secondary tRNA structure (**b**,**d**,**f**). Significance cutoff limit is shown as a red line in (**a**,**c**,**e**)—see text for discussion.

## References

[1] Schimmel P. (2008). Development of tRNA synthetases and connection to genetic code and disease. Protein Sci..

[2] Giegé R., Eriani G. (2014). Transfer RNA Recognition and Aminoacylation by Synthetases. eLS.

[3] Eriani G., Karam J., Jacinto J., Richard E.M., Geslain R. (2015). MIST, a Novel Approach to Reveal Hidden Substrate Specificity in Aminoacyl-tRNA Synthetases. PLoS ONE.

[4] Cvetesic N., Gruic-Sovulj I. (2017). Synthetic and editing reactions of aminoacyl-tRNA synthetases using cognate and non-cognate amino acid substrates. Methods.

[5] Sapienza P., Li L., Williams T., Lee A., Carter C.W. (2016). An Ancestral Tryptophanyl-tRNA Synthetase Precursor Achieves High Catalytic Rate Enhancement without Ordered Ground-State Tertiary Structures. ACS Chem. Biol..

[6] Martinez-Rodriguez L., Erdogan O., Jimenez-Rodriguez M., Gonzalez-Rivera K., Williams T., Li L., Weinreb V., Collier M., Chandrasekaran S., Ambroggio X. (2015). Functional Class I and II Amino Acid-activating Enzymes Can Be Coded by Opposite Strands of the Same Gene. J. Biol. Chem..

[7] Li R., Macnamara L., Leuchter J., Alexander R., Cho S. (2015). MD Simulations of tRNA and Aminoacyl-tRNA Synthetases: Dynamics, Folding, Binding, and Allostery. Int. J. Mol. Sci..

[8] Adrion J., White P., Montooth K. (2016). The Roles of Compensatory Evolution and Constraint in Aminoacyl tRNA Synthetase Evolution. Mol. Biol. Evol..

[9] Fang P., Guo M. (2017). Structural characterization of human aminoacyl-tRNA synthetases for translational and nontranslational functions. Methods.

[10] Datt M., Sharma A. (2014). Novel and unique domains in aminoacyl-tRNA synthetases from human fungal pathogens Aspergillus niger, Candida albicans and Cryptococcus neoformans. BMC Genom..

[11] Amiram M., Haimovich A., Fan C., Wang Y., Aerni H., Ntai I., Moonan D., Ma N., Rovner A., Hong S. (2015). Evolution of translation machinery in recoded bacteria enables multi-site incorporation of nonstandard amino acids. Nat. Biotechnol..

[12] Terasaka N., Iwane Y., Geiermann A., Goto Y., Suga H. (2015). Recent developments of engineered translational machineries for the incorporation of non-canonical amino acids into polypeptides. Int. J. Mol. Sci..

[13] Perli E., Fiorillo A., Giordano C., Pisano A., Montanari A., Grazioli P., Campese A., Di Micco P., Tuppen H., Genovese I. (2016). Short peptides from leucyl-tRNA synthetase rescue disease-causing mitochondrial tRNA point mutations. Hum. Mol. Genet..

[14] Rodin A., Gogoshin G., Litvinenko A., Boerwinkle E., Rao C., Chakraborty R. (2012). Exploring genetic epidemiology data with Bayesian networks. Handbook of Statistics.

[15] Gogoshin G., Boerwinkle E., Rodin A. (2017). New Algorithm and Software (BNOmics) for Inferring and Visualizing Bayesian Networks from Heterogeneous Big Biological and Genetic Data. J. Comput. Biol..

[16] Juhling F., Morl M., Hartmann R., Sprinzl M., Stadler P., Putz J. (2009). tRNAdb 2009: Compilation of tRNA sequences and tRNA genes. Nucleic Acids Res..

[17] Quigley G., Rich A. (1976). Structural domains of transfer RNA molecules. Science.

[18] Heckerman D. (1995). A Tutorial on Learning with Bayesian Networks.

[19] Pearl J. (2009). Causality: Models, Reasoning, and Inference.

[20] Zhang X., Branciamore S., Gogoshin G., Rodin A.S., Riggs A.D. (2017). Analysis of high-resolution 3D intrachromosomal interactions aided by Bayesian network modeling. Proc. Natl. Acad. Sci. USA.

[21] Kullback S., Leibler R.A. (1951). On information and sufficiency. Ann. Math. Stat..

[22] Lin J. (2002). Divergence measures based on the Shannon entropy. IEEE Trans. Inf. Theory.

[23] Sprinzl M., Horn C., Brown M., Ioudovitch A., Steinberg S. (1998). Compilation of tRNA sequences and sequences of tRNA genes. Nucleic Acids Res..

[24] Laslett D., Canback B. (2004). ARAGORN, a program to detect tRNA genes and tmRNA genes in nucleotide sequences. Nucleic Acids Res..

[25] Giegé R., Sissler M., Florentz C. (1998). Universal rules and idiosyncratic features in tRNA identity. Nucleic Acids Res..

[26] Galili T., Gingold H., Shaul S., Benjamini Y. (2016). Identifying the ligated amino acid of archaeal tRNAs based on positions outside the anticodon. RNA.

[27] Zamudio G., Jose M. (2017). Identity Elements of tRNA as Derived from Information Analysis. Orig. Life Evol. Biosph..

[28] Rodin A., Litvinenko A., Klos K., Morrison A., Woodage T., Coresh J., Boerwinkle E. (2009). Use of wrapper algorithms coupled with a random forests classifier for variable selection in large-scale genomic association studies. J. Comput. Biol..

[29] Saffarian A., Giraud M., Touzet H. (2015). Modeling alternate RNA structures in genomic sequences. J. Comput. Biol..

[30] Mustoe A., Liu X., Lin P., Al-Hashimi H., Fierke C., Brooks C.L. (2015). Noncanonical secondary structure stabilizes mitochondrial tRNA(Ser(UCN)) by reducing the entropic cost of tertiary folding. J. Am. Chem. Soc..

[31] Velandia-Huerto C., Berkemer S., Hoffmann A., Retzlaff N., Marroquin L.C.R., Hernandez-Rosales M., Stadler P., Bermudez-Santana C. (2016). Orthologs, turn-over, and remolding of tRNAs in primates and fruit flies. BMC Genom..

[32] Schimmel P. (2018). The emerging complexity of the tRNA world: Mammalian tRNAs beyond protein synthesis. Nat. Rev. Mol. Cell Biol..

[33] Koonin E., Novozhilov A. (2017). Origin and Evolution of the Universal Genetic Code. Annu. Rev. Genet..

[34] Carter C.W., Wills P. (2017). Interdependence, Reflexivity, Fidelity, Impedance Matching, and the Evolution of Genetic Coding. Mol. Biol. Evol..

[35] Wills P., Carter C.W. (2017). Insuperable problems of the genetic code initially emerging in an RNA world. Biosystems.

[36] Di Giulio M. (2017). The aminoacyl-tRNA synthetases had only a marginal role in the origin of the organization of the genetic code: Evidence in favor of the coevolution theory. J. Theor. Biol..

[37] Di Giulio M. (2017). Some pungent arguments against the physico-chemical theories of the origin of the genetic code and corroborating the coevolution theory. J. Theor. Biol..

