# Supplementary material for: Intrinsic Properties of tRNA Molecules as Deciphered via Bayesian Network and Distribution Divergence Analysis
Source: Life (Basel). 2018 Feb 8;8(1):5. doi: 10.3390/life8010005 (PMC5871937; doi:10.3390/life8010005)
Supplement: Supplementary file 1 [file life-08-00005-s001.pdf]

Article

Intrinsic properties of tRNA molecules as deciphered via Bayesian Network and distribution divergence analysis.

Sergio Branciamore [1] *, Grigoriy Gogoshin [1], Massimo Di Giulio [2] and Andrei S. Rodin [1], *

[1] Department of Diabetes Complications and Metabolism, Diabetes and Metabolism Research Institute, City of Hope, Duarte, CA, USA
[2] Early Evolution of Life Laboratory, Institute of Biosciences and Bioresources, CNR,Naples, Italy
* Correspondence: sbranciamore@coh.org; arodin@coh.org

Academic Editor: name
Version February 6, 2018 submitted to MDPI

0. Supplementary Informations

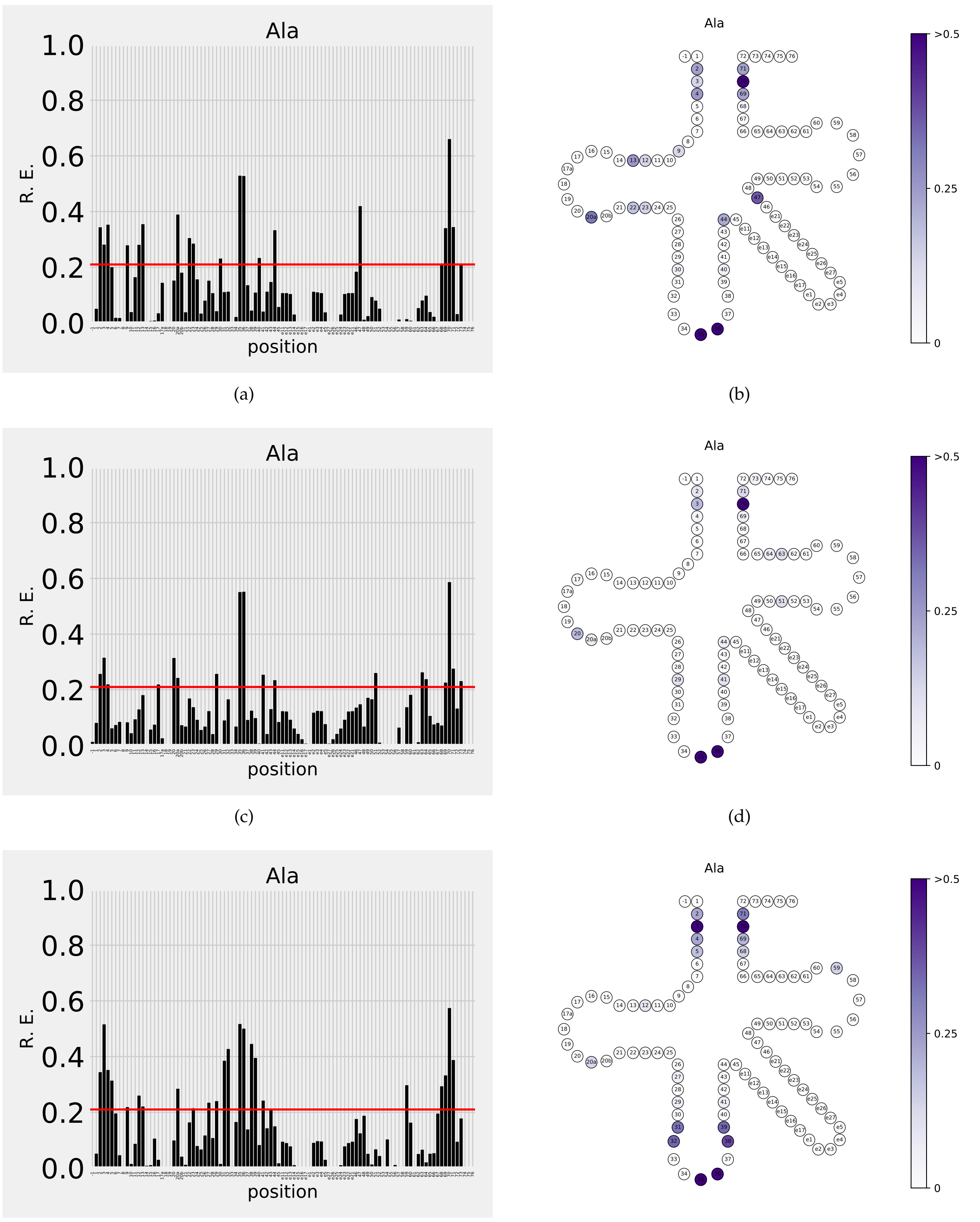

(a) (b)

(c) (d)

(e) (f)

Figure 1. Position "importance" profile forAla tRNAs. (a,b) Archea, (c,d) Bacteria, (e,f) Eukaria. Relative Entropy is shown as function of tRNA position (enumerated as in Figure 1, first row). Significance cutoff limit is shown as a red line (see text for discussion).

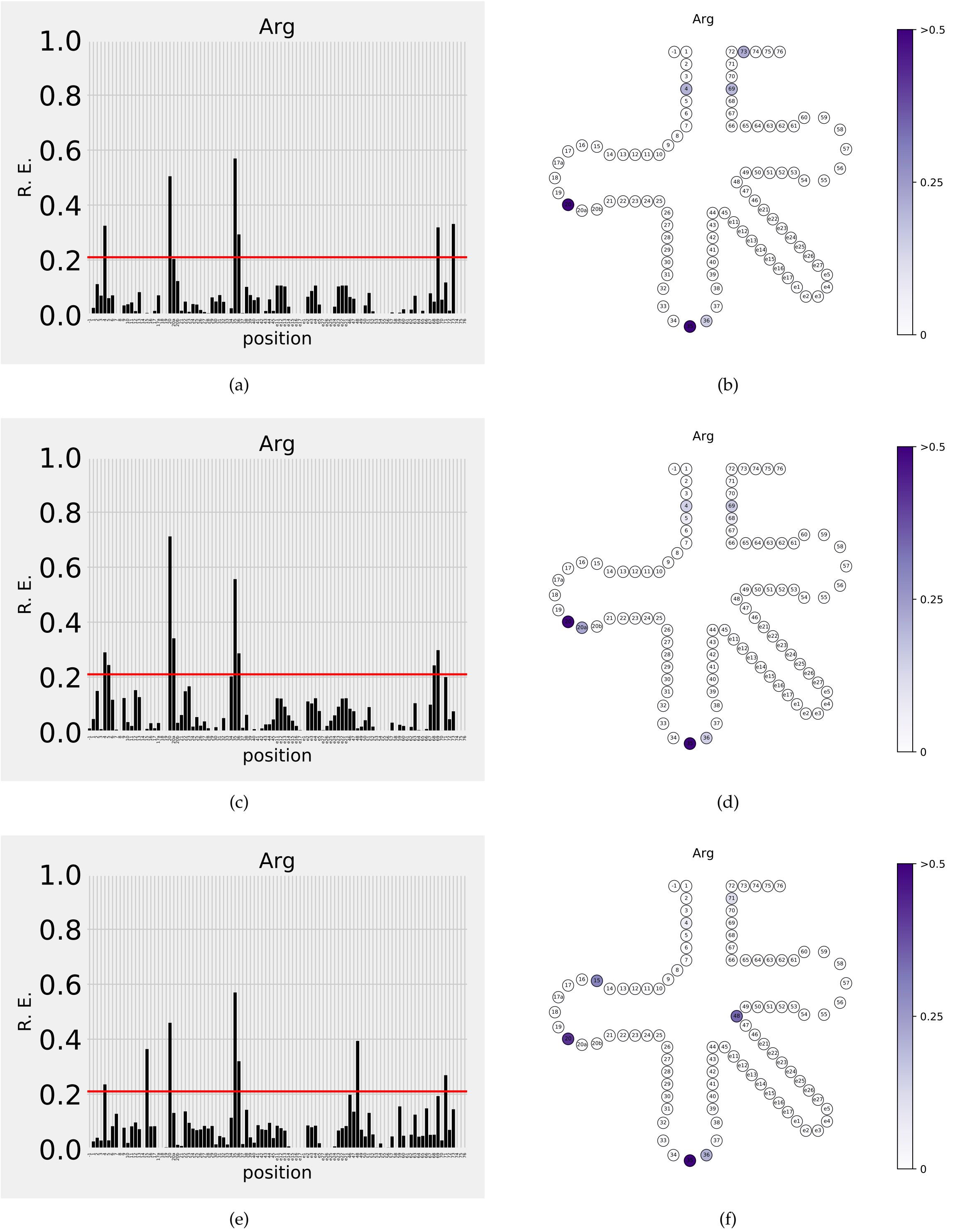

Figure 2. Position "importance" profile forArg tRNAs. (a,b) Archea, (c,d) Bacteria, (e,f) Eukaria. Relative Entropy is shown as function of tRNA position (enumerated as in Figure 1, first row). Significance cutoff limit is shown as a red line (see text for discussion).

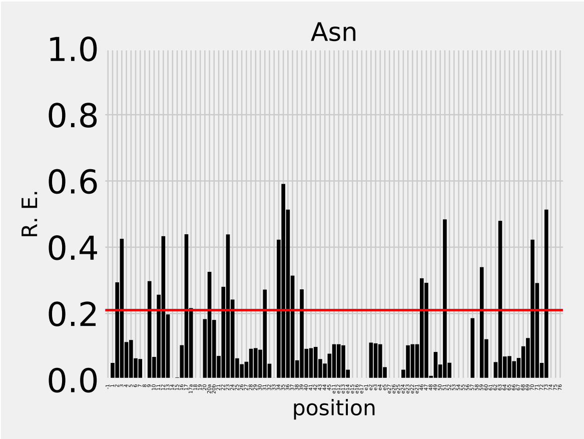

(a)

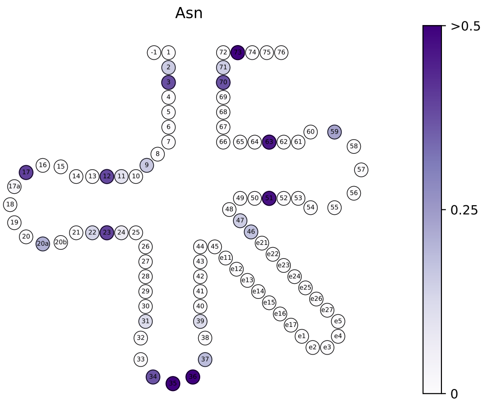

(b)

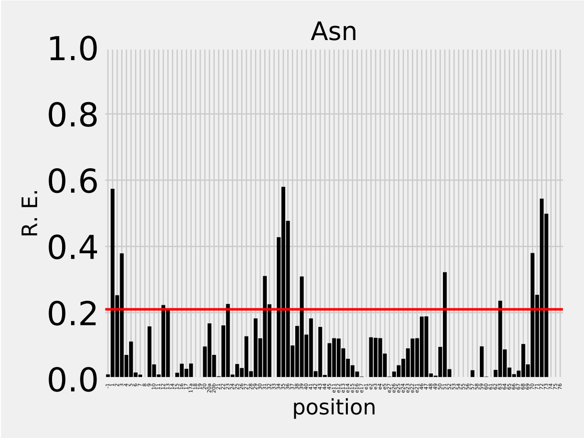

(c)

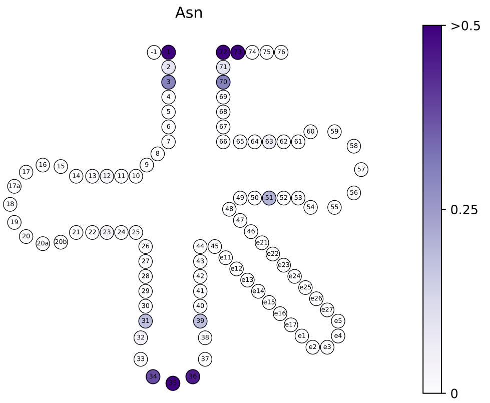

(d)

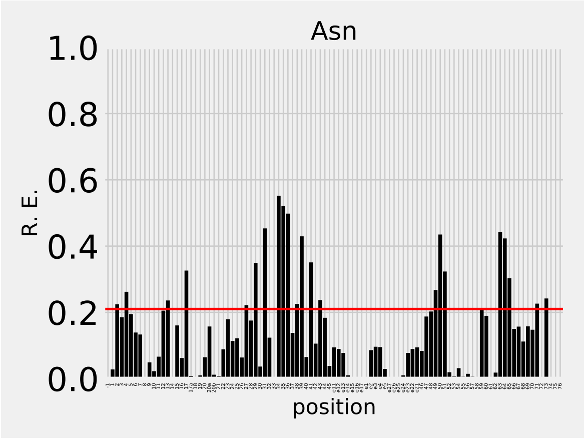

(e)

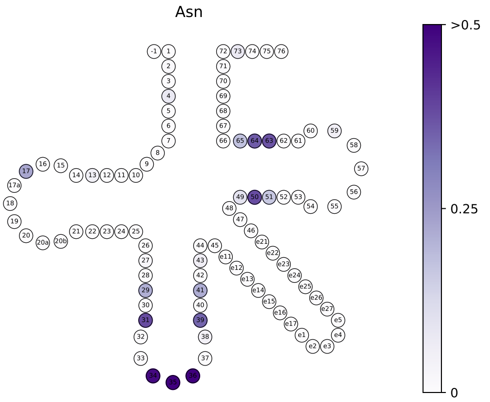

(f)

Figure 3. Position "importance" profile forAsn tRNAs. (a,b) Archea, (c,d) Bacteria, (e,f) Eukaria. Relative Entropy is shown as function of tRNA position (enumerated as in Figure 1, first row). Significance cutoff limit is shown as a red line (see text for discussion).

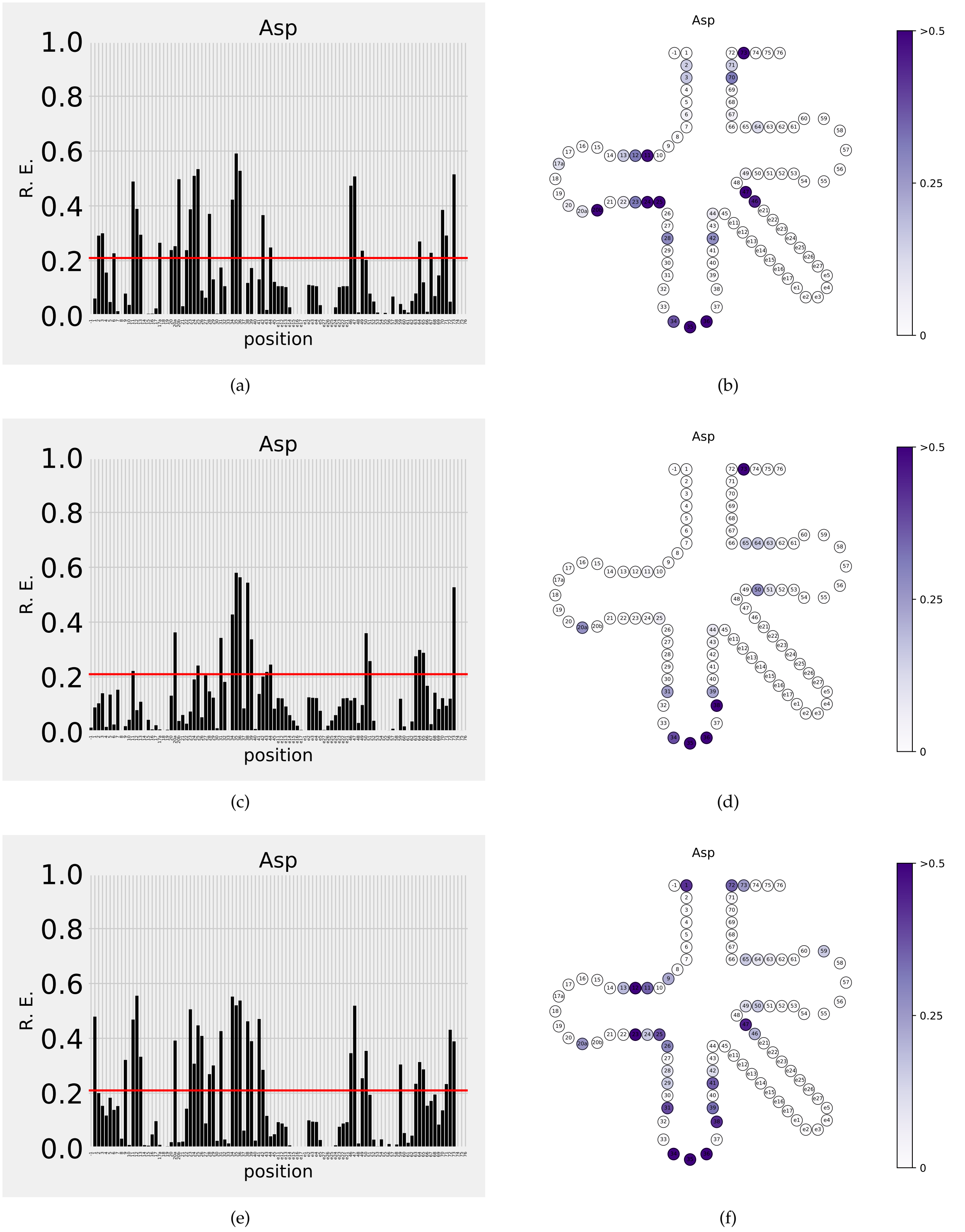

Figure 4. Position "importance" profile forAsp tRNAs. (a,b) Archea, (c,d) Bacteria, (e,f) Eukaria. Relative Entropy is shown as function of tRNA position (enumerated as in Figure 1, first row). Significance cutoff limit is shown as a red line (see text for discussion).

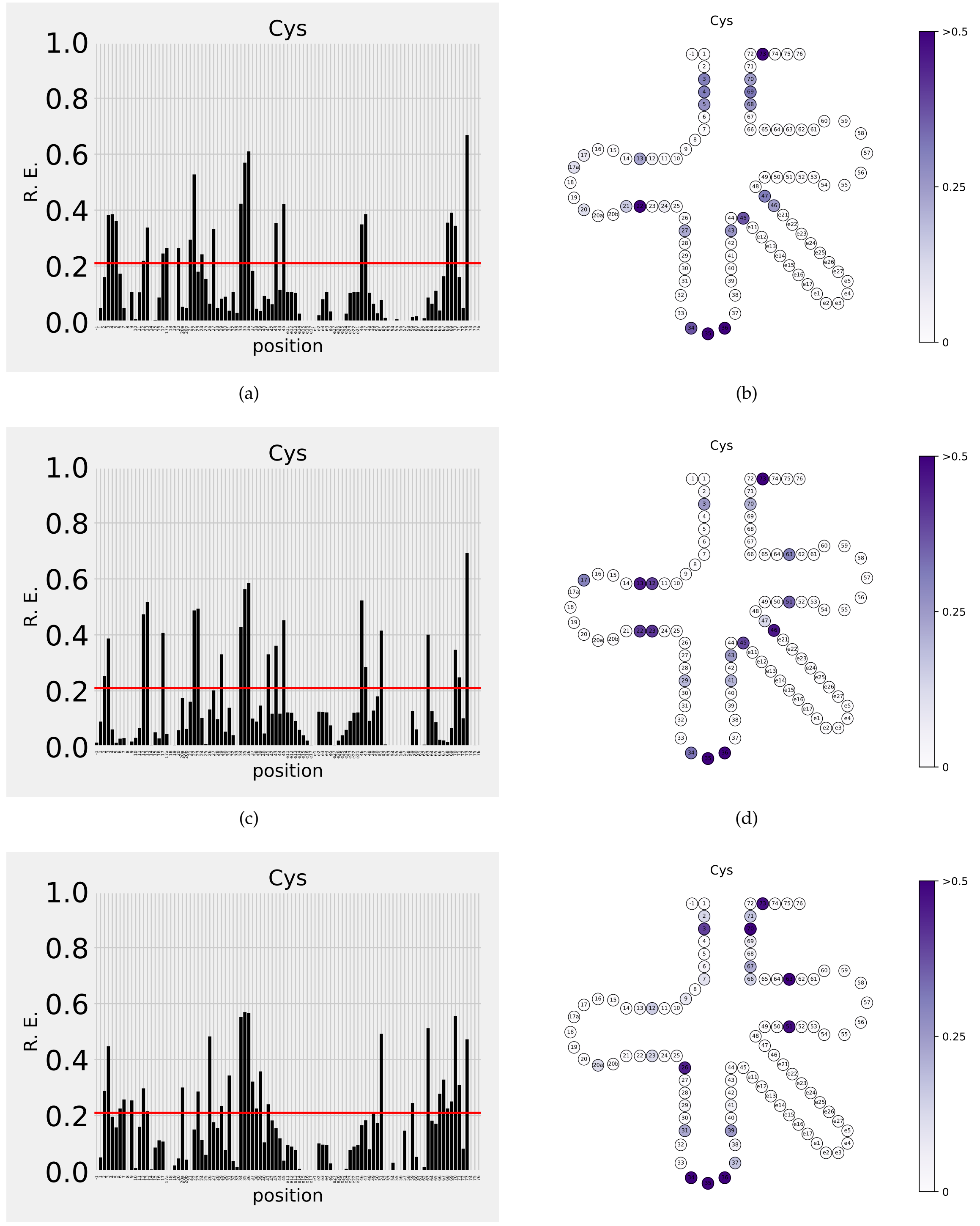

(a) (b)

(c) (d)

(e) (f)

Figure 5. Position "importance" profile forCys tRNAs. (a,b) Archea, (c,d) Bacteria, (e,f) Eukaria. Relative Entropy is shown as function of tRNA position (enumerated as in Figure 1, first row). Significance cutoff limit is shown as a red line (see text for discussion).

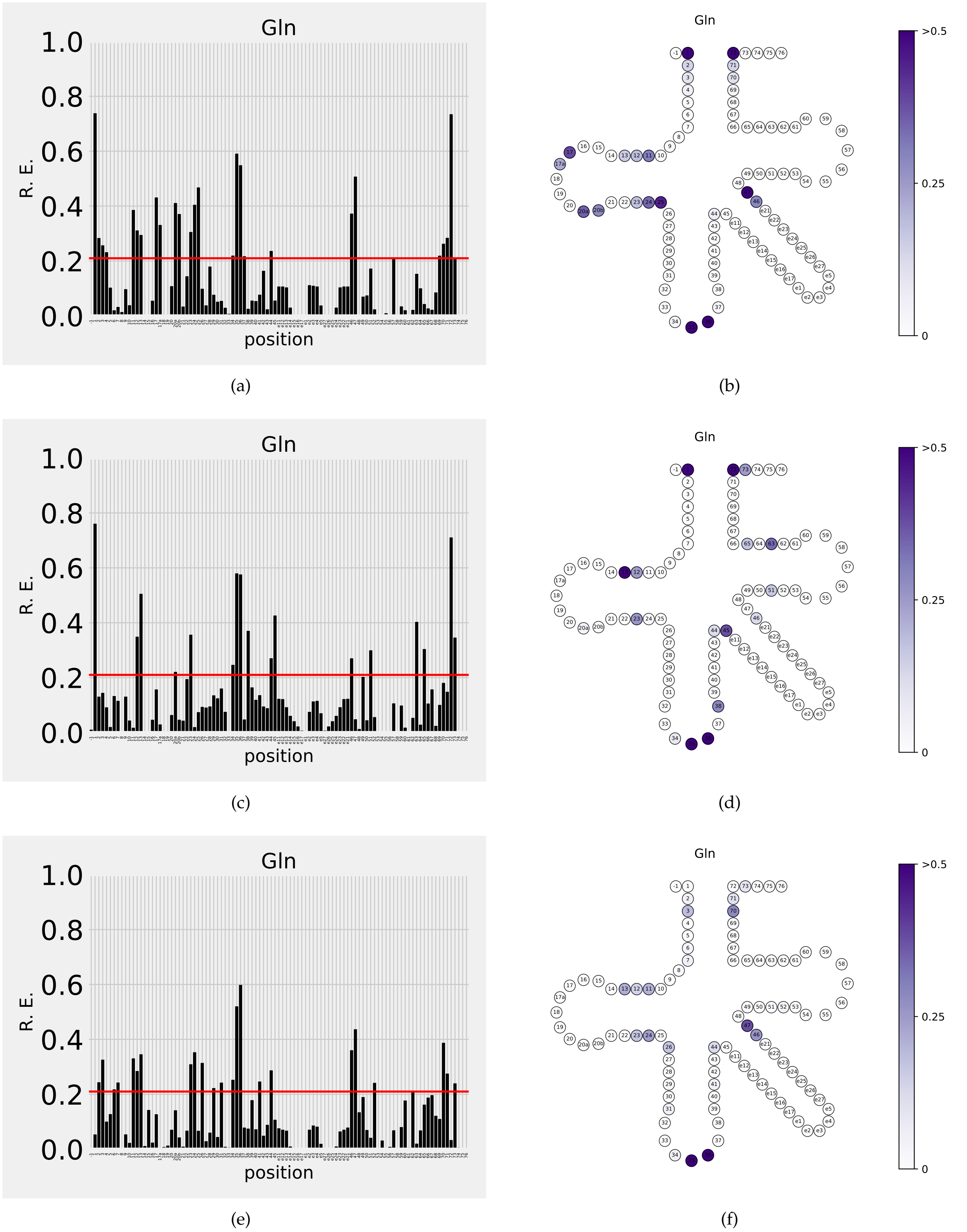

Figure 6. Position "importance" profile forGln tRNAs. (a,b) Archea, (c,d) Bacteria, (e,f) Eukaria. Relative Entropy is shown as function of tRNA position (enumerated as in Figure 1, first row). Significance cutoff limit is shown as a red line (see text for discussion).

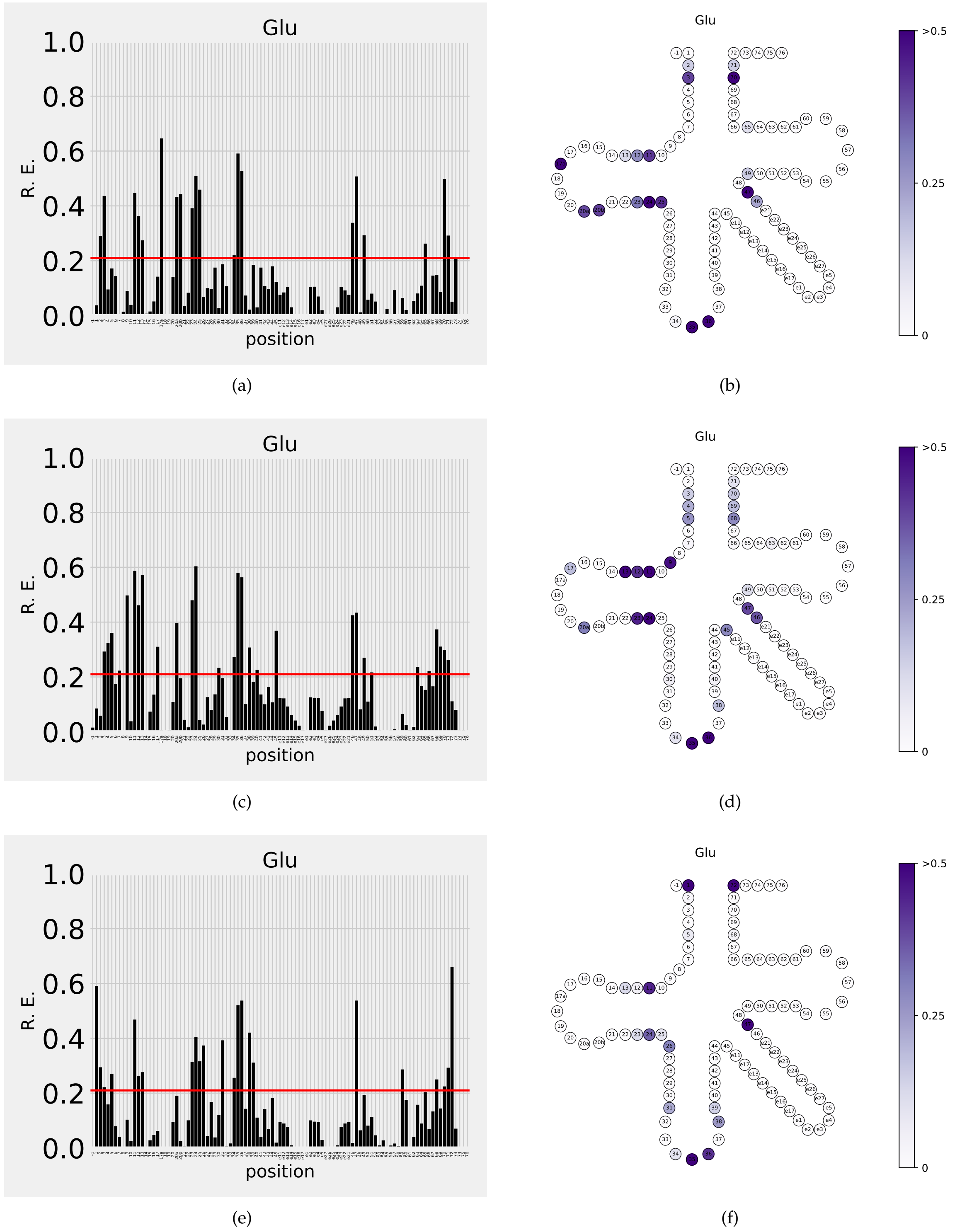

Figure 7. Position "importance" profile forGlu tRNAs. (a,b) Archea, (c,d) Bacteria, (e,f) Eukaria. Relative Entropy is shown as function of tRNA position (enumerated as in Figure 1, first row). Significance cutoff limit is shown as a red line (see text for discussion).

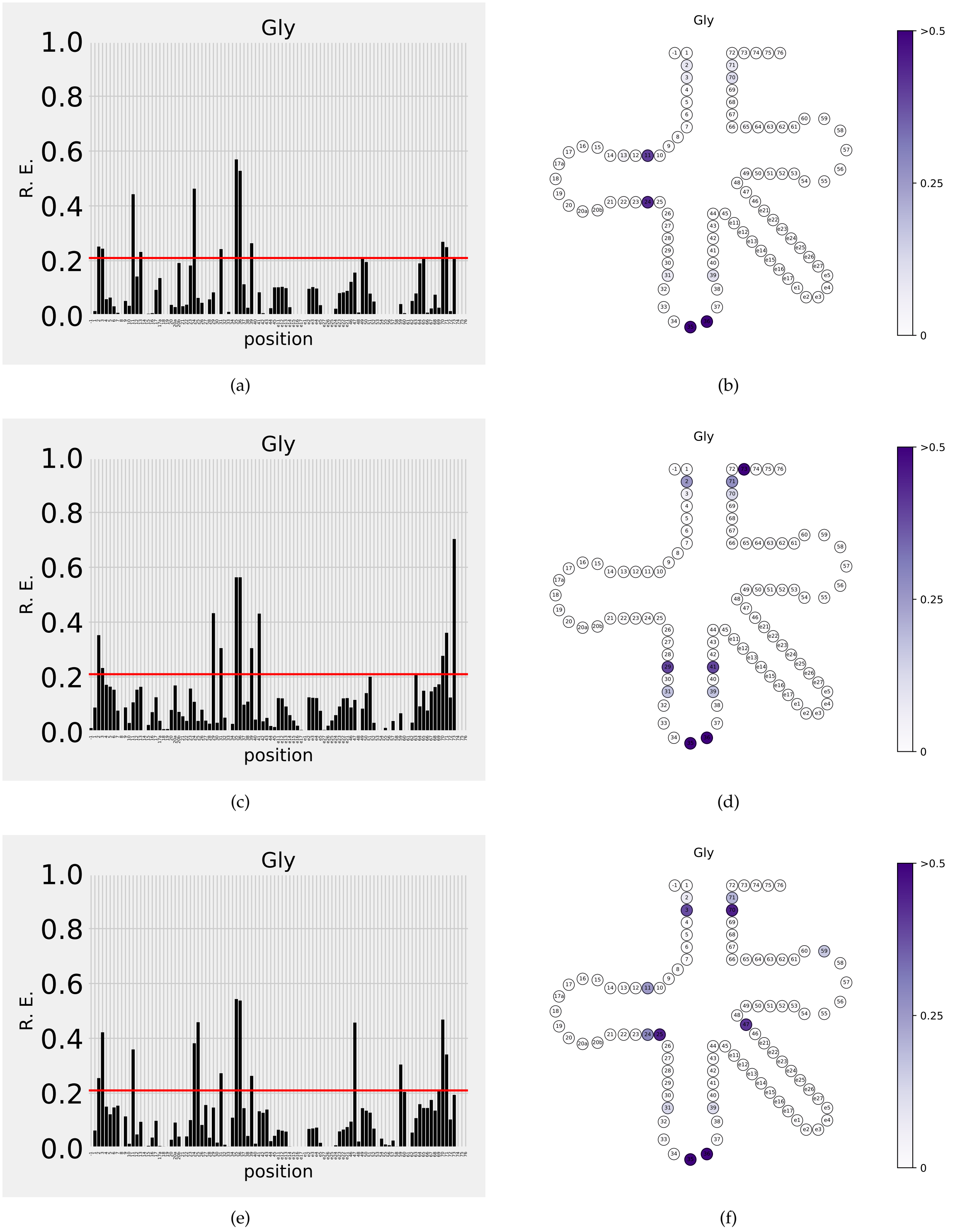

Figure 8. Position "importance" profile forGly tRNAs. (a,b) Archea, (c,d) Bacteria, (e,f) Eukaria. Relative Entropy is shown as function of tRNA position (enumerated as in Figure 1, first row). Significance cutoff limit is shown as a red line (see text for discussion).

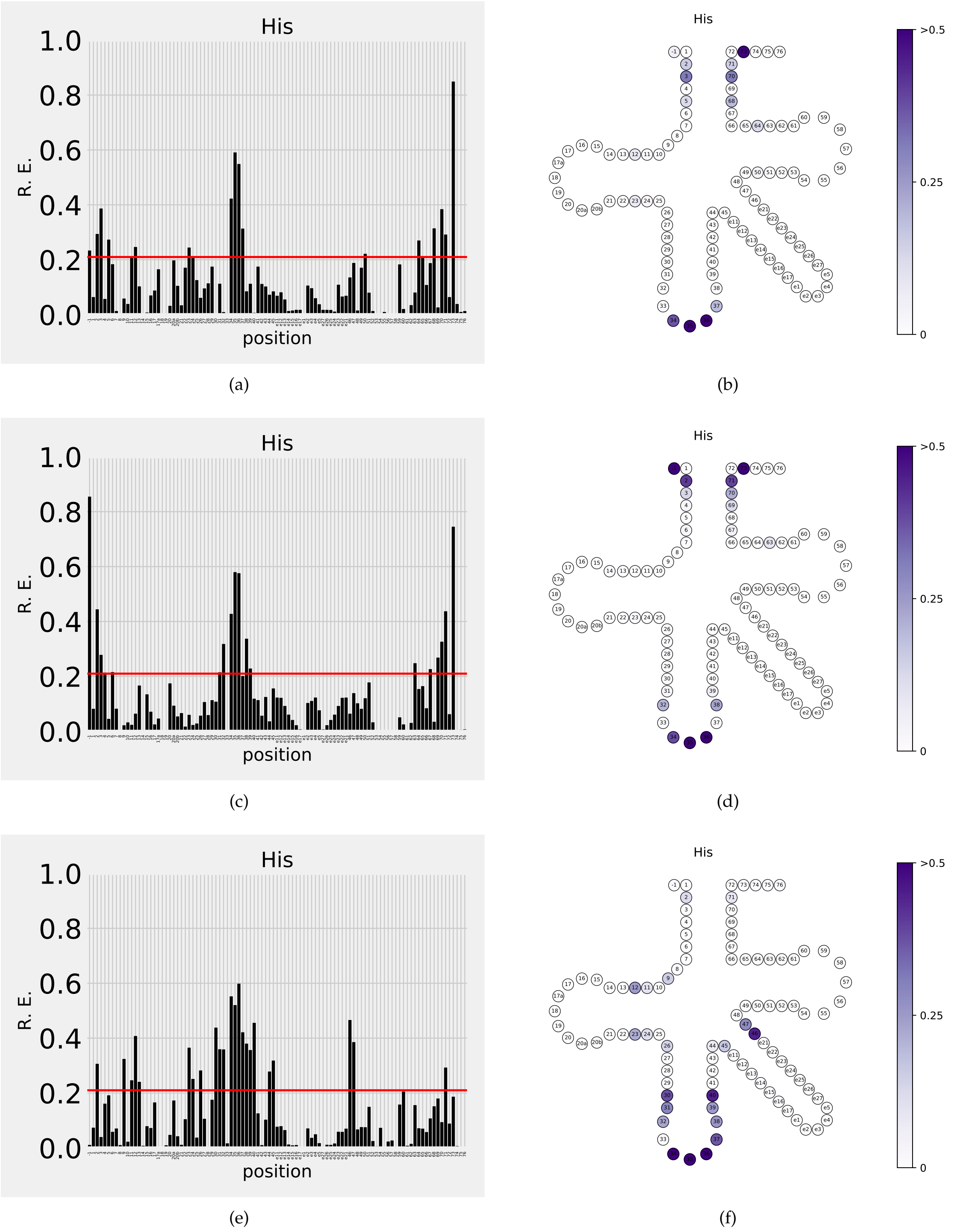

Figure 9. Position "importance" profile forHis tRNAs. (a,b) Archea, (c,d) Bacteria, (e,f) Eukaria. Relative Entropy is shown as function of tRNA position (enumerated as in Figure 1, first row). Significance cutoff limit is shown as a red line (see text for discussion).

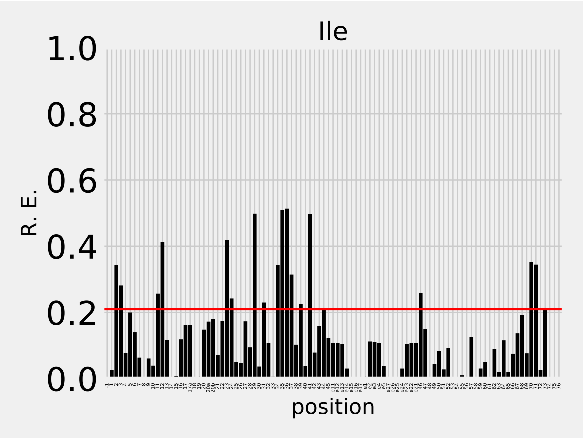

(a)

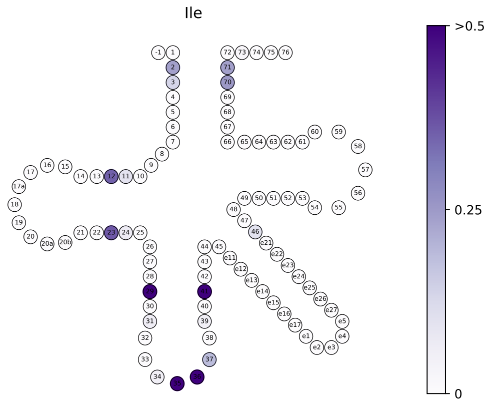

(b)

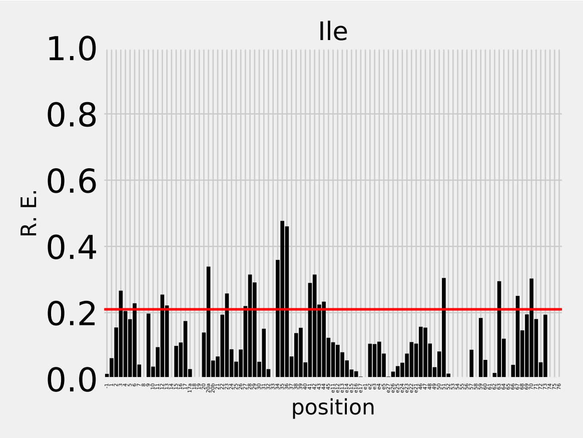

(c)

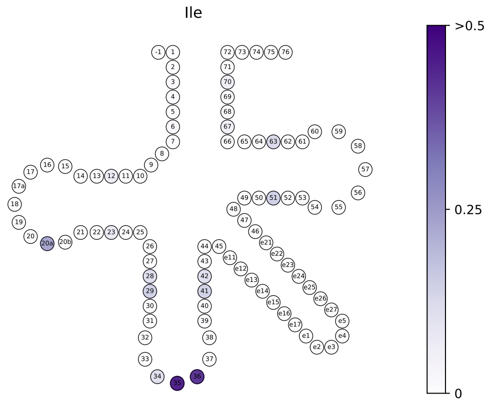

(d)

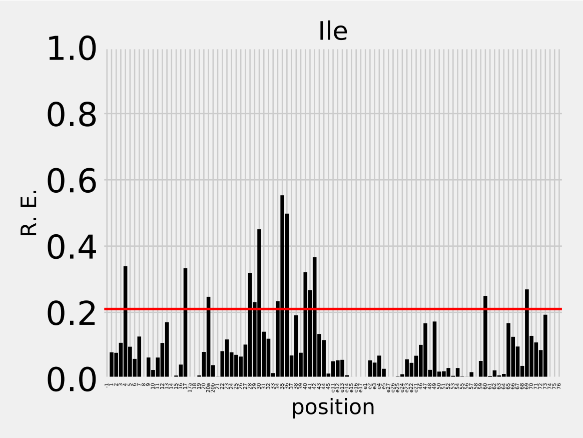

(e)

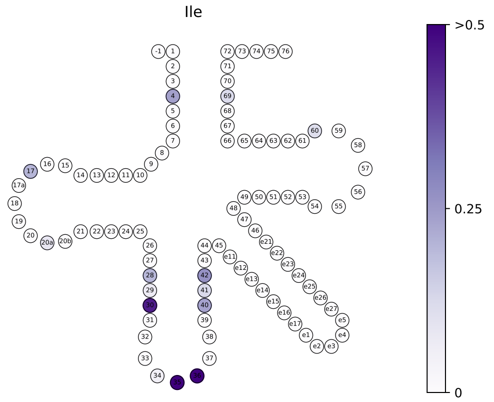

(f)

Figure 10. Position "importance" profile forIle tRNAs. (a,b) Archea, (c,d) Bacteria, (e,f) Eukaria. Relative Entropy is shown as function of tRNA position (enumerated as in Figure 1, first row). Significance cutoff limit is shown as a red line (see text for discussion).

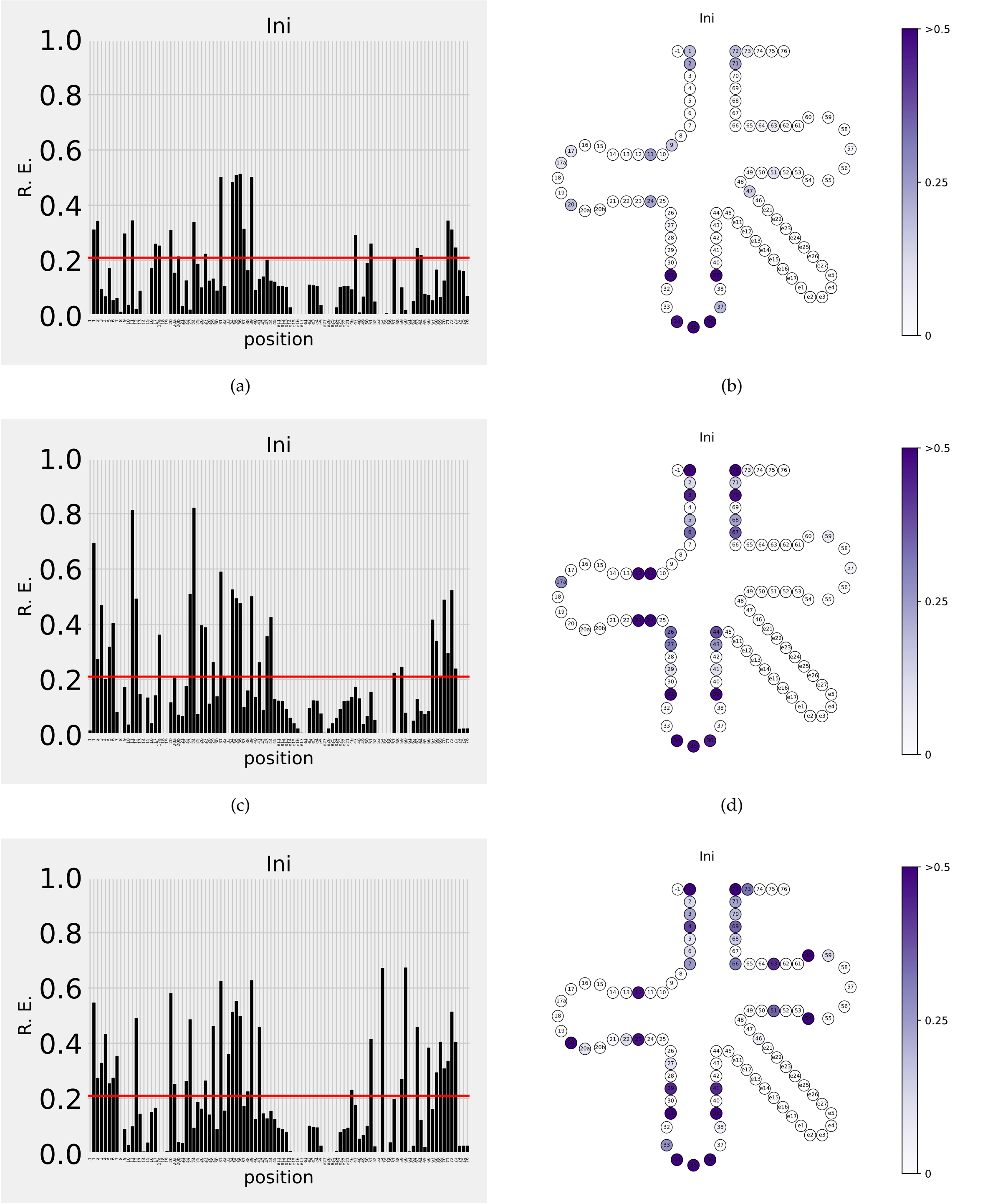

(a) (b)

(c) (d)

(e) (f)

Figure 11. Position "importance" profile forIni tRNAs. (a,b) Archea, (c,d) Bacteria, (e,f) Eukaria. Relative Entropy is shown as function of tRNA position (enumerated as in Figure 1, first row). Significance cutoff limit is shown as a red line (see text for discussion).

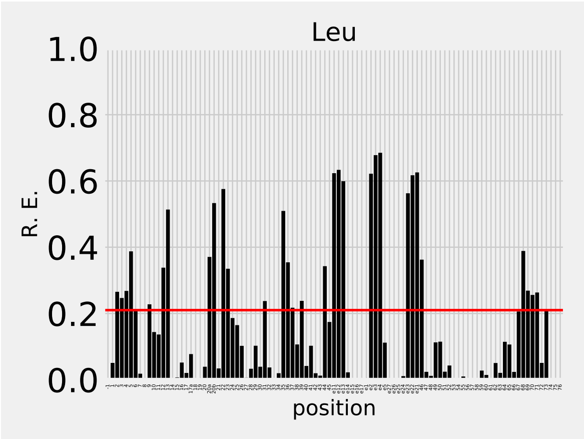

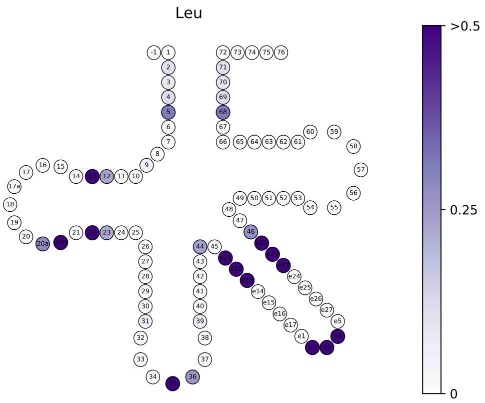

(a) (b)

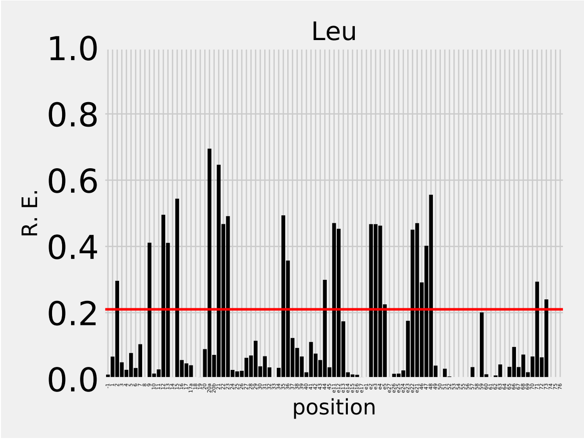

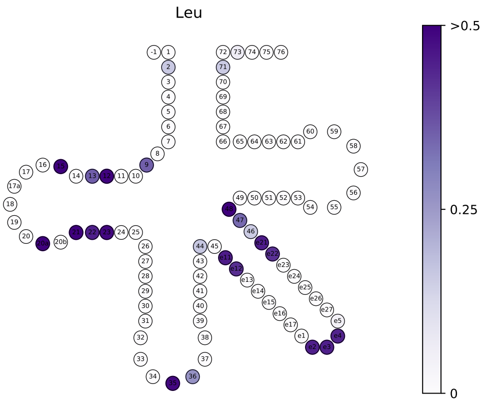

(c) (d)

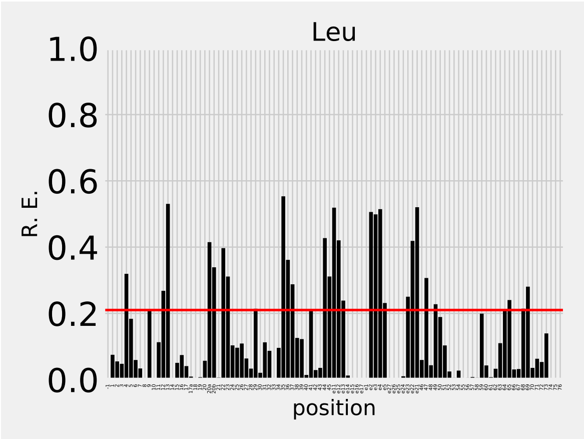

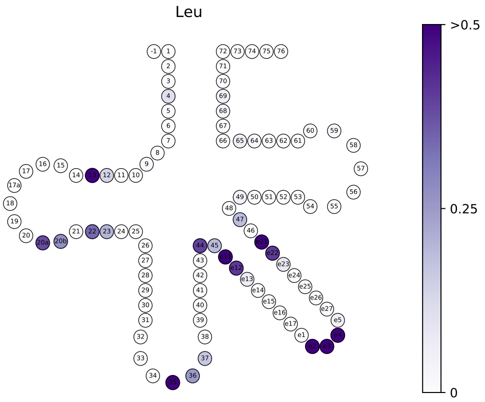

(e) (f)

Figure 12. Position "importance" profile forLeu tRNAs. (a,b) Archea, (c,d) Bacteria, (e,f) Eukaria. Relative Entropy is shown as function of tRNA position (enumerated as in Figure 1, first row). Significance cutoff limit is shown as a red line (see text for discussion).

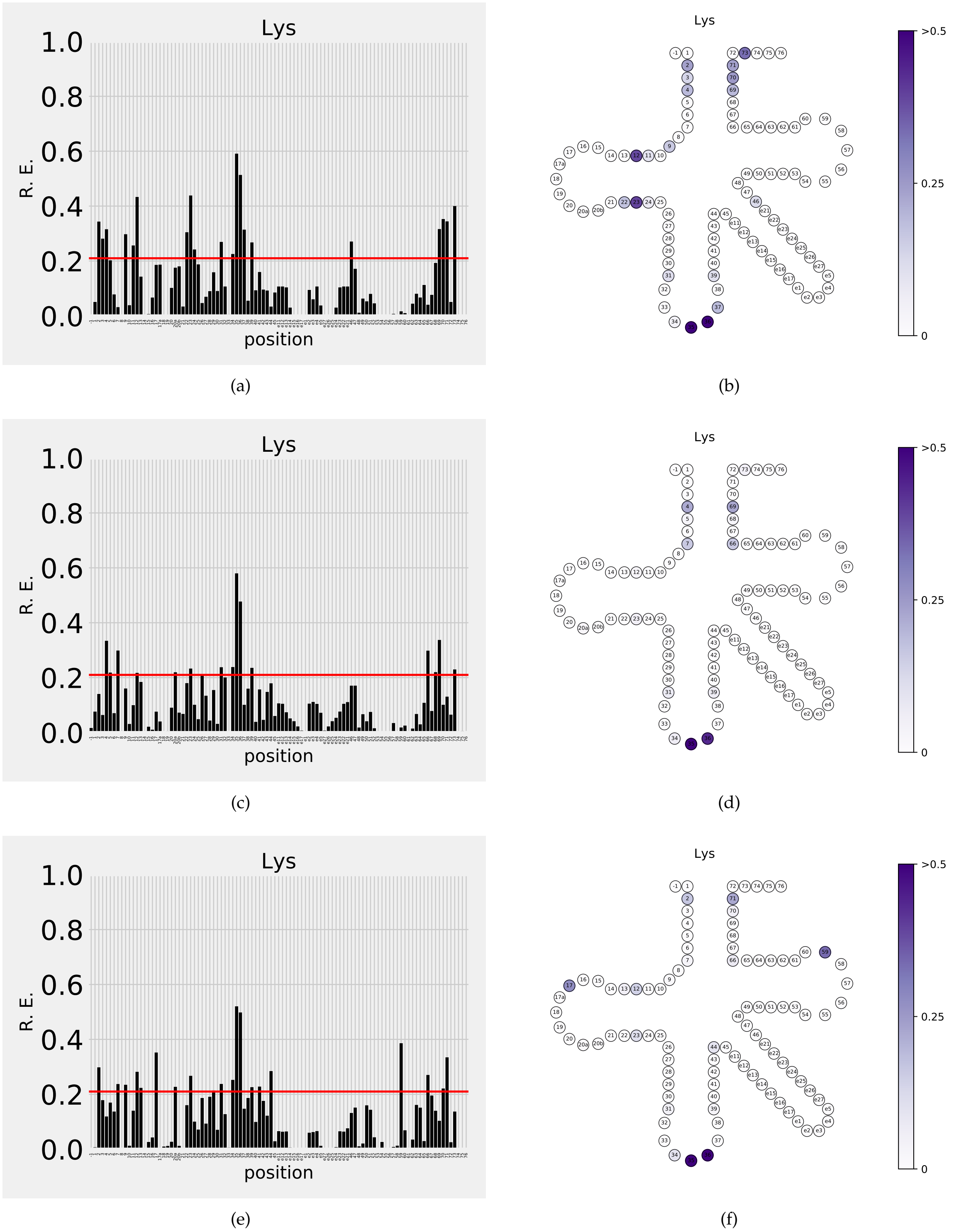

Figure 13. Position "importance" profile forLys tRNAs. (a,b) Archea, (c,d) Bacteria, (e,f) Eukaria. Relative Entropy is shown as function of tRNA position (enumerated as in Figure 1, first row). Significance cutoff limit is shown as a red line (see text for discussion).

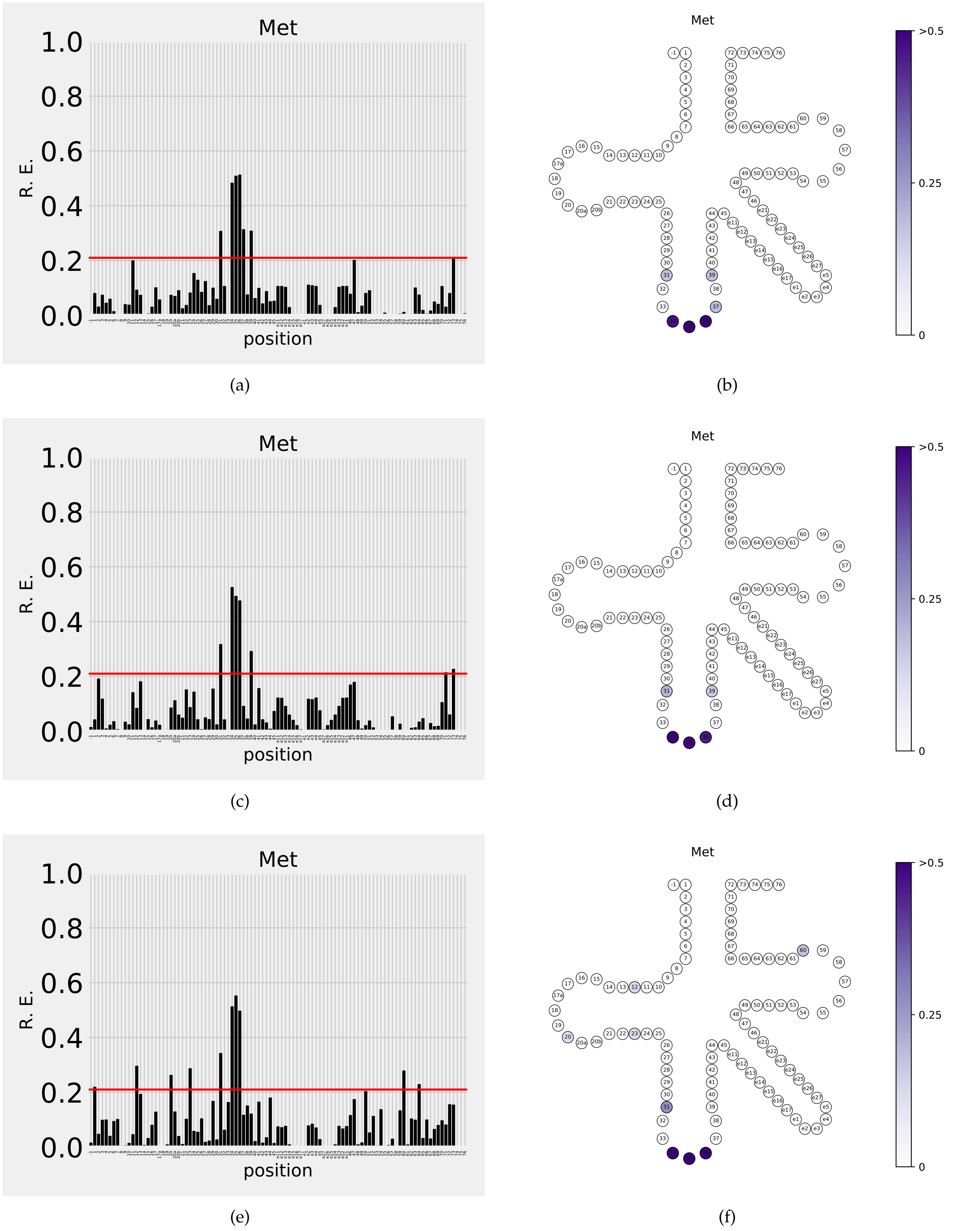

Figure 14. Position "importance" profile forMet tRNAs. (a,b) Archea, (c,d) Bacteria, (e,f) Eukaria. Relative Entropy is shown as function of tRNA position (enumerated as in Figure 1, first row). Significance cutoff limit is shown as a red line (see text for discussion).

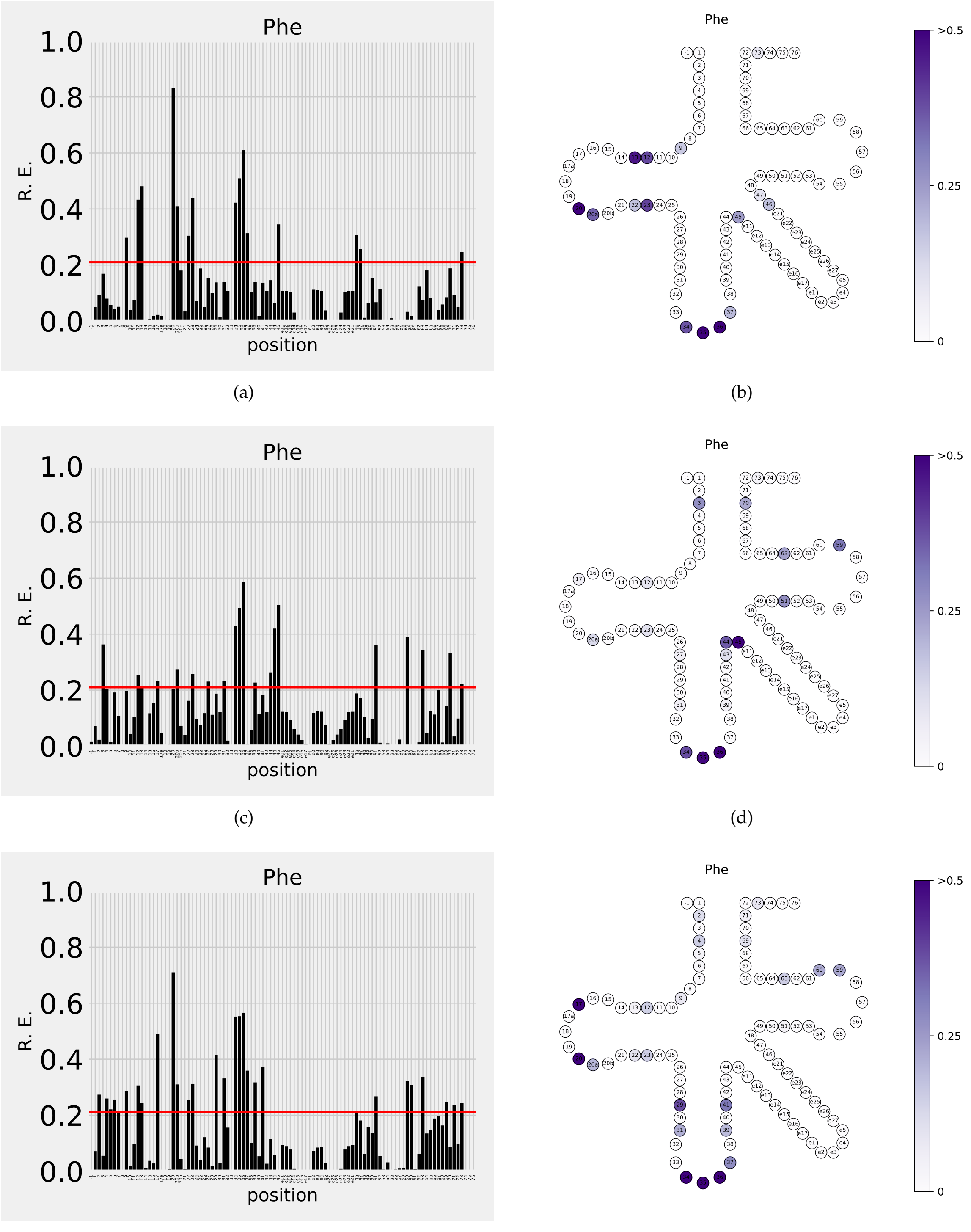

Figure 15. Position "importance" profile forPhe tRNAs. (a,b) Archea, (c,d) Bacteria, (e,f) Eukaria. Relative Entropy is shown as function of tRNA position (enumerated as in Figure 1, first row). Significance cutoff limit is shown as a red line (see text for discussion).

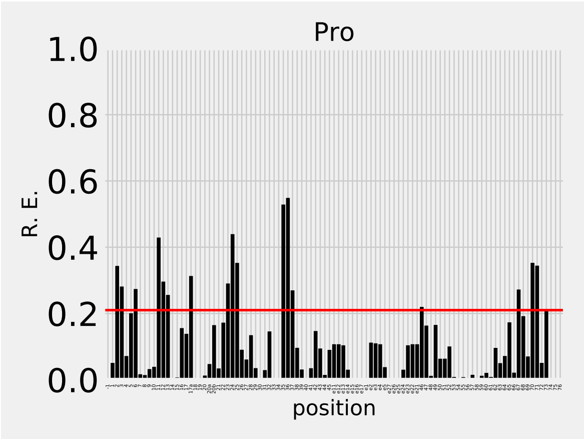

(a)

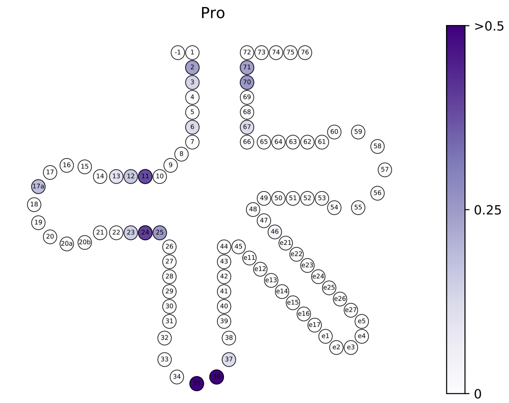

(b)

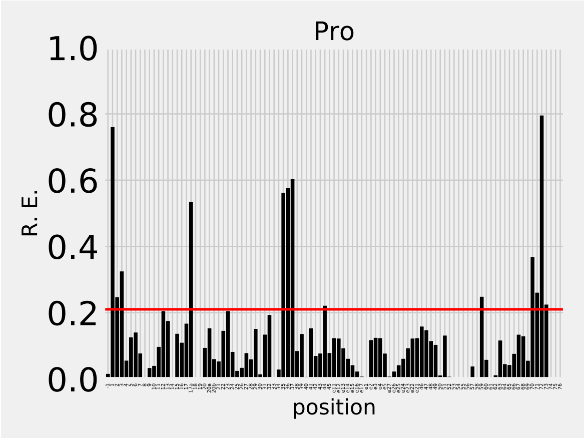

(c)

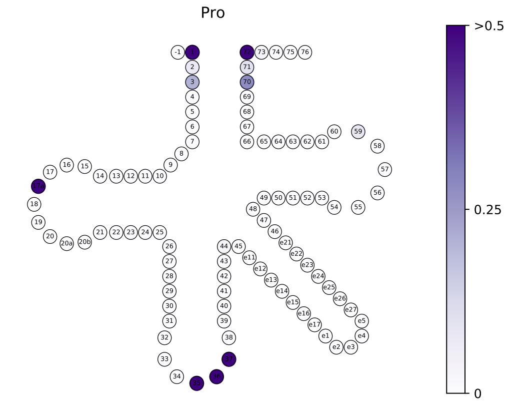

(d)

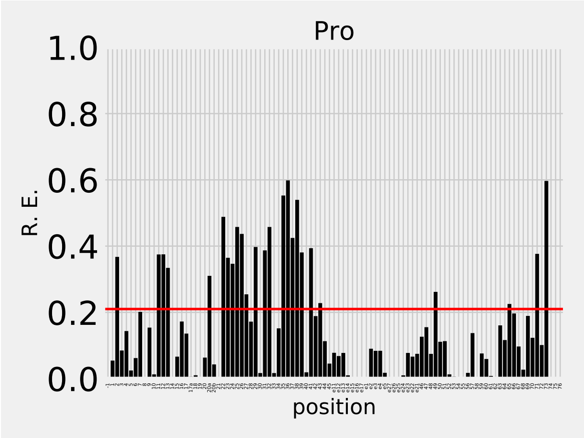

(e)

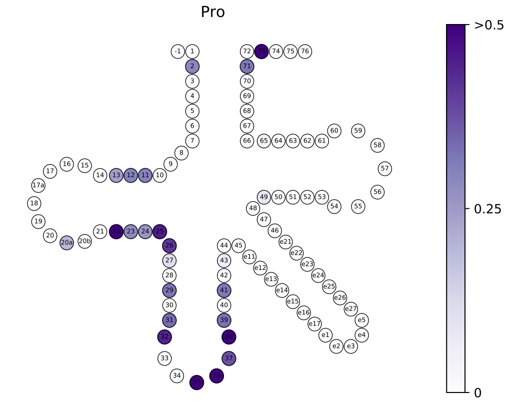

(f)

Figure 16. Position “importance” profile forPro tRNAs. (a,b) Archea, (c,d) Bacteria, (e,f) Eukaria. Relative Entropy is shown as function of tRNA position (enumerated as in Figure 1, first row). Significance cutoff limit is shown as a red line (see text for discussion).

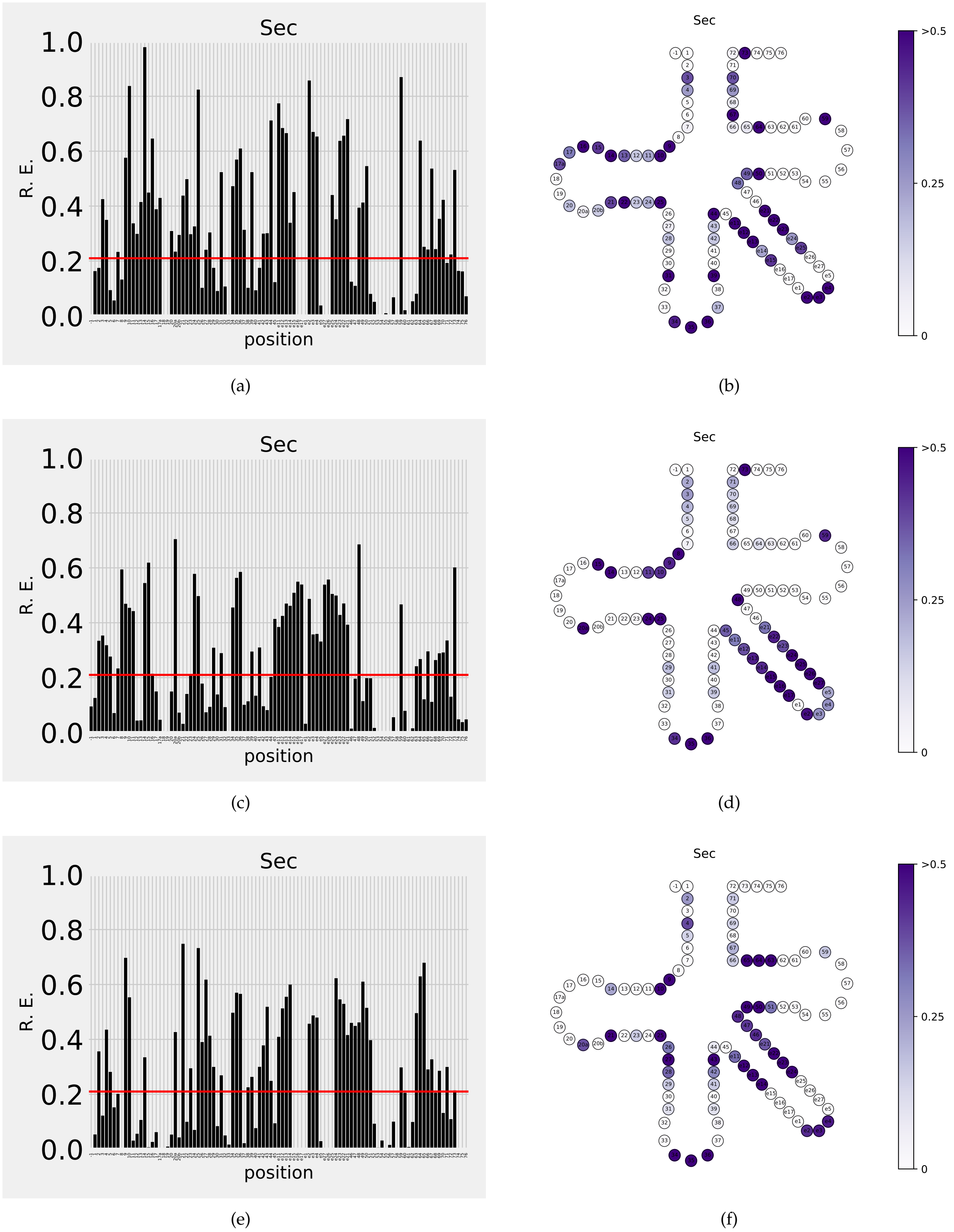

Figure 17. Position "importance" profile forSec tRNAs. (a,b) Archea, (c,d) Bacteria, (e,f) Eukaria. Relative Entropy is shown as function of tRNA position (enumerated as in Figure 1, first row). Significance cutoff limit is shown as a red line (see text for discussion).

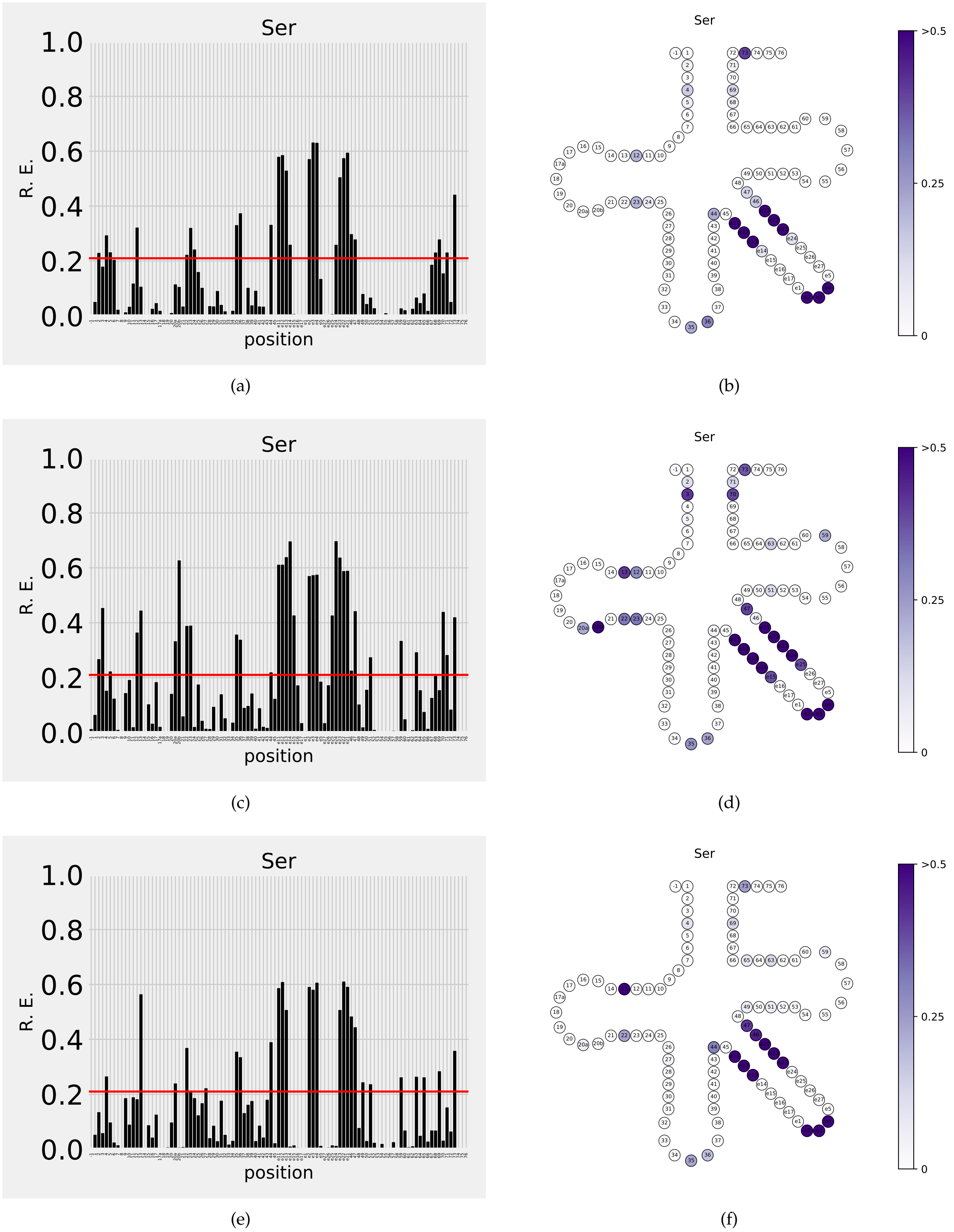

(a) (b)

(c) (d)

(e) (f)

Figure 18. Position "importance" profile forSer tRNAs. (a,b) Archea, (c,d) Bacteria, (e,f) Eukaria. Relative Entropy is shown as function of tRNA position (enumerated as in Figure 1, first row). Significance cutoff limit is shown as a red line (see text for discussion).

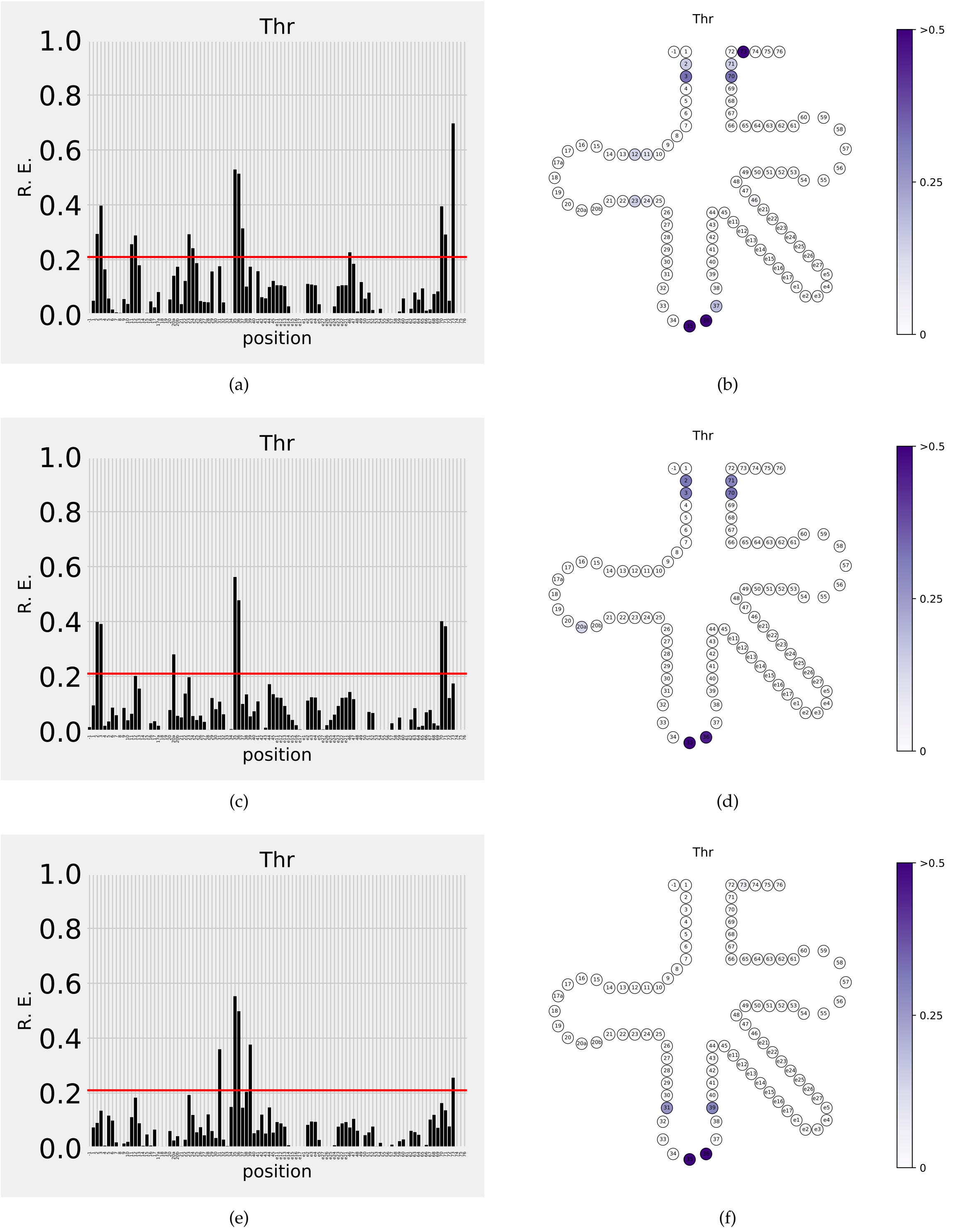

(a) (b)

(c) (d)

(e) (f)

Figure 19. Position "importance" profile forThr tRNAs. (a,b) Archea, (c,d) Bacteria, (e,f) Eukaria. Relative Entropy is shown as function of tRNA position (enumerated as in Figure 1, first row). Significance cutoff limit is shown as a red line (see text for discussion).

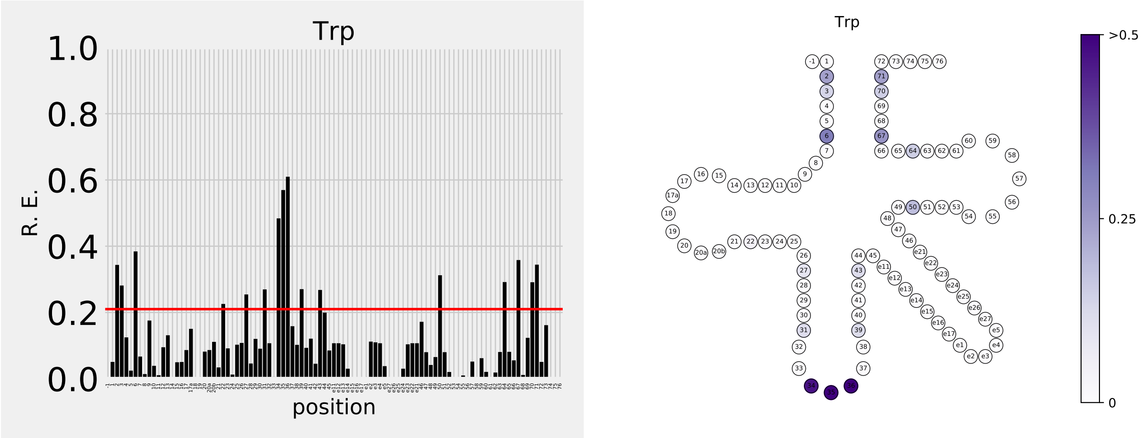

(a) (b)

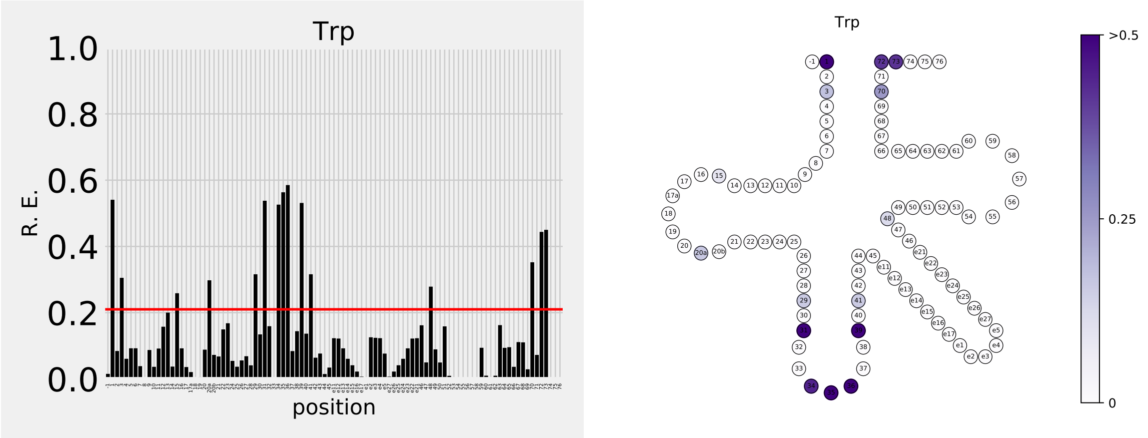

(c) (d)

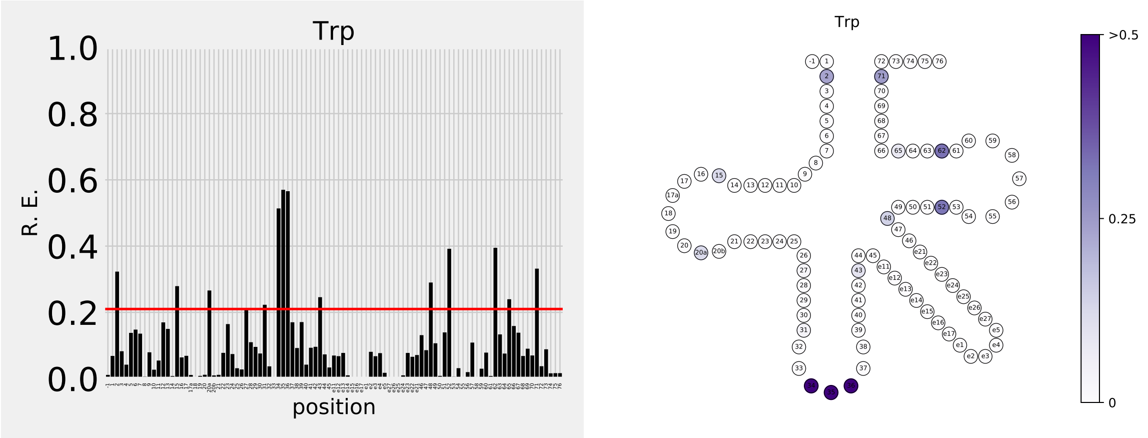

(e) (f)

Figure 20. Position "importance" profile forTrp tRNAs. (a,b) Archea, (c,d) Bacteria, (e,f) Eukaria. Relative Entropy is shown as function of tRNA position (enumerated as in Figure 1, first row). Significance cutoff limit is shown as a red line (see text for discussion).

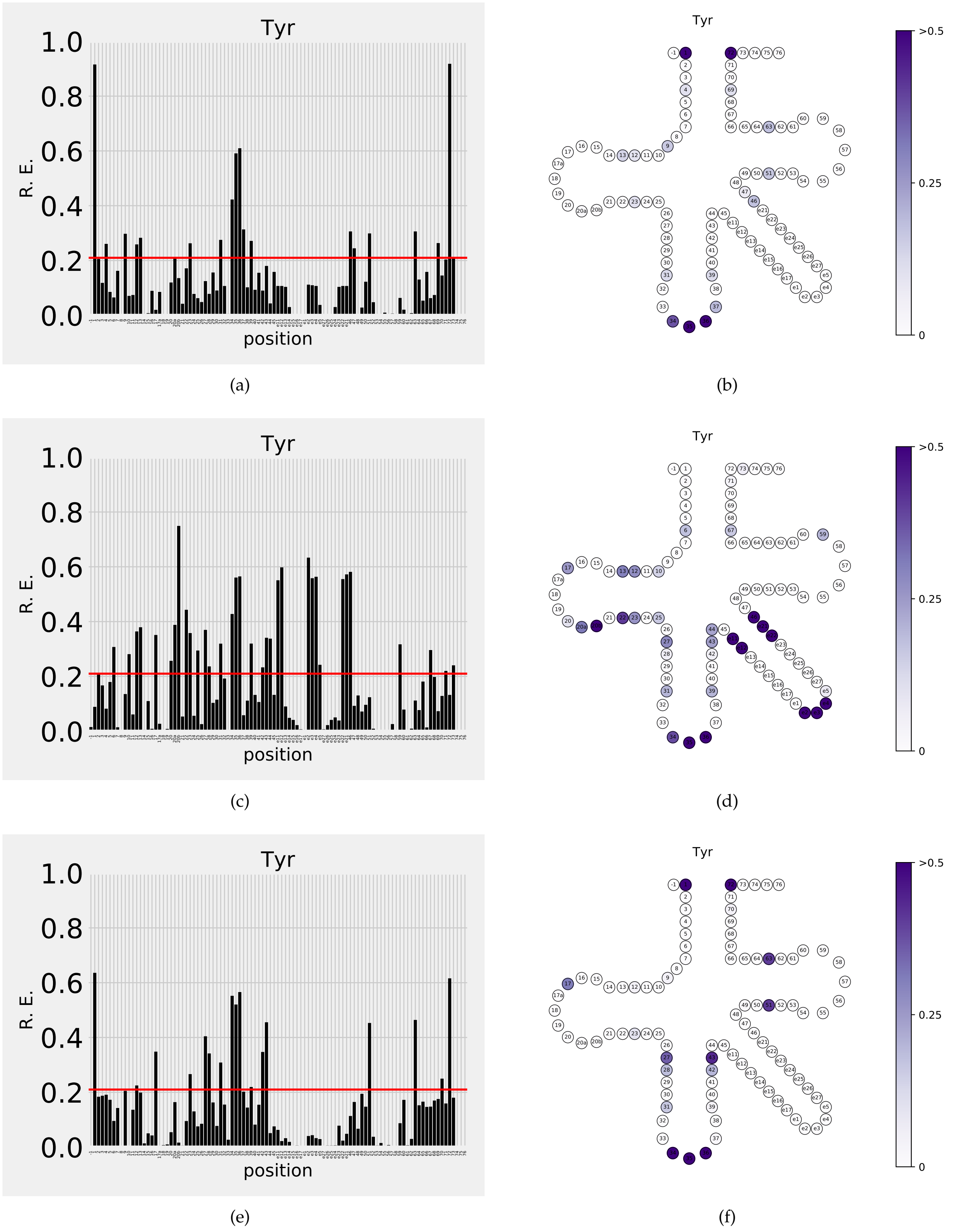

Figure 21. Position “importance” profile forTyr tRNAs. (a,b) Archea, (c,d) Bacteria, (e,f) Eukaria. Relative Entropy is shown as function of tRNA position (enumerated as in Figure 1, first row). Significance cutoff limit is shown as a red line (see text for discussion).

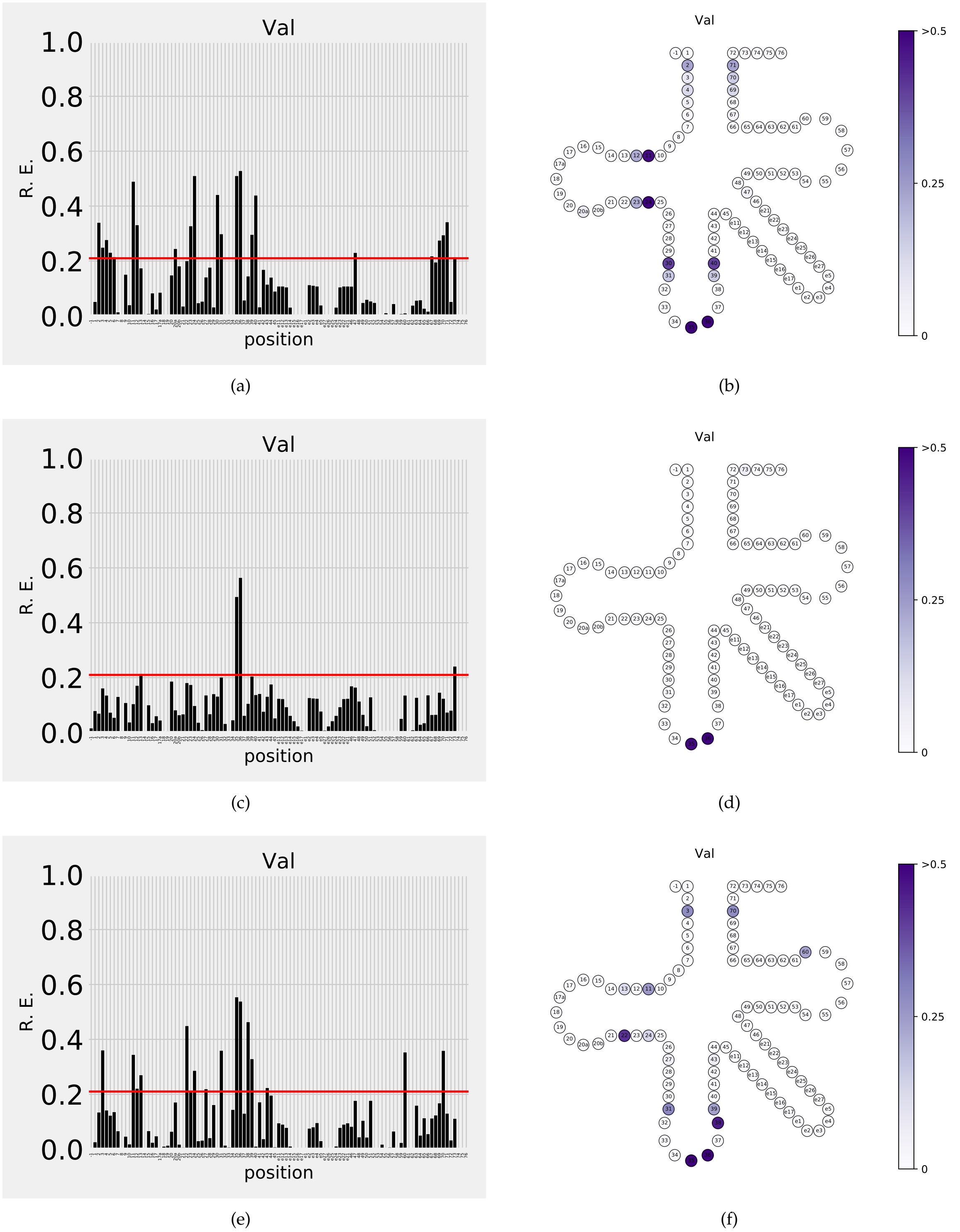

Figure 22. Position "importance" profile forVal tRNAs. (a,b) Archea, (c,d) Bacteria, (e,f) Eukaria. Relative Entropy is shown as function of tRNA position (enumerated as in Figure 1, first row). Significance cutoff limit is shown as a red line (see text for discussion).

© 2018 by the authors. Submitted to *MDPI* for possible open access publication under the terms and conditions of the Creative Commons Attribution (CC BY) license (http://creativecommons.org/licenses/by/4.0/).
